# Better Safe than Sorry - Socio-Spatial Group Structure Emerges from Individual Variation in Fleeing, Avoidance or Velocity in an Agent-Based Model

**DOI:** 10.1371/journal.pone.0026189

**Published:** 2011-11-18

**Authors:** Ellen Evers, Han de Vries, Berry M. Spruijt, Elisabeth H. M. Sterck

**Affiliations:** Behavioural Biology, Utrecht University, Utrecht, The Netherlands; Georgia State University, United States of America

## Abstract

In group-living animals, such as primates, the average spatial group structure often reflects the dominance hierarchy, with central dominants and peripheral subordinates. This central-peripheral group structure can arise by self-organization as a result of subordinates fleeing from dominants after losing a fight. However, in real primates, subordinates often avoid interactions with potentially aggressive group members, thereby preventing aggression and subsequent fleeing. Using agent-based modeling, we investigated which spatial and encounter structures emerge when subordinates also avoid known potential aggressors at a distance as compared with the model which only included fleeing after losing a fight (fleeing model). A central-peripheral group structure emerged in most conditions. When avoidance was employed at small or intermediate distances, centrality of dominants emerged similar to the fleeing model, but in a more pronounced way. This result was also found when fleeing after a fight was made independent of dominance rank, i.e. occurred randomly. Employing avoidance at larger distances yielded more spread out groups. This provides a possible explanation of larger group spread in more aggressive species. With avoidance at very large distances, spatially and socially distinct subgroups emerged. We also investigated how encounters were distributed amongst group members. In the fleeing model all individuals encountered all group members equally often, whereas in the avoidance model encounters occurred mostly among similar-ranking individuals. Finally, we also identified a very general and simple mechanism causing a central-peripheral group structure: when individuals merely differed in velocity, faster individuals automatically ended up at the periphery. In summary, a central-peripheral group pattern can easily emerge from individual variation in different movement properties in general, such as fleeing, avoidance or velocity. Moreover, avoidance behavior also affects the encounter structure and can lead to subgroup formation.

## Introduction

Understanding animal behavior within its social context remains a challenge, since individuals are situated in a complex social environment that consists of many interacting entities and is typically structured, both spatially and socially [1]–[4]. A socio-spatial group structure (or pattern) describes how social and spatial properties of individuals, e.g. dominance rank and spatial position within the group, relate to each other [5], [6]. In this paper we aim to identify some general mechanisms, which generate socio-spatial group structures. Our theoretical results, obtained via agent-based modeling, are applicable to group-living species, where the dominance hierarchy plays a prominent role in determining the spatial group structure. Here, we will mainly focus on primates and relate our findings to empirical primate studies.

A particular socio-spatial structure often reported in primate studies is a central-peripheral group pattern, i.e. dominant individuals are at the center of the group and subordinates populate the periphery (macaques: [7]–[16], capuchins: [17]–[19], baboons: [20]–[22]). Concerning this centrality of dominants, different theories have been put forward to explain how or why this group pattern may come about. From an ultimate point of view, individuals may have evolved an instinctive preference for the central position within the group to lower predation risk (‘selfish herd theory’, [23]). On the proximate level, this spatial preference may cause dominants to monopolize this preferred central position. Another, purely social, proximate explanation suggests that the average spatial pattern is a side-effect of the movements of all individuals relative to each other. By means of an agent-based model called DomWorld, Hemelrijk showed that aggressive dominance interactions and subsequent fleeing by the loser gives rise to a central-peripheral spatial structure [5]. This spatial pattern emerged through self-organization, meaning that the model individuals had no preference for any spatial location whatsoever. Such a cognitively minimalistic proximate mechanism is likely to extend far beyond the primates.

The crucial element in Hemelrijk's model is the flight of the loser after an aggressive encounter. However, in contrast to these model entities, real primates often try to reduce or prevent aggression within a group, which results in less aggressive encounters and less fleeing. To reduce negative consequences of aggressive conflicts, primates follow numerous strategies either during or after the conflict, e.g. fleeing, policing, redirection or post-conflict affiliation [24]–[31]. However, aggression may also simply be prevented in the first place through conflict avoidance [32]–[34]. When maneuvering within the group, individuals can adjust their spatial position with respect to potential aggressors to reduce the chance of encounters and the ensuing risk of receiving aggression [17], [27], [35]–[43]. Primate species can be characterized by their dominance style [44]. Vehrencamp distinguished between egalitarian and despotic styles [45] and Thierry suggested a whole continuum of dominance styles [46]. Despotic species are characterized by a steeper dominance hierarchy, more unidirectional aggression, higher variance of within-group aggressiveness, lower levels of tolerance and a more pronounced centrality of dominants compared to egalitarian species. Avoidance of potential aggressors is especially important in aggressive, intolerant species and in species that lack formal submission signals [27], [31], [36], [39], such as patas monkeys [42], [47]–[49]. Especially in species with a despotic dominance hierarchy, the rank distance between two individuals is reflected in avoidance behavior [37], [50]. Avoidance behavior is therefore likely to be an important determinant of spatial structuring within a group, however, researchers have not yet investigated this specific potential role of avoidance behavior.

We integrated avoidance behavior into an established model framework to investigate its effect on the socio-spatial properties of a group of individuals. To study this we constructed an agent-based model. Agent-based models (ABMs, also called individual-based models or IBM) are especially helpful to systematically study and understand the structuring mechanisms in a complex system [51]–[53]. Whereas empirical results from behavioral observations and experiments provide the ingredients for a theoretical model, ABMs can complement and provide feedback on this empirical research itself and on the underlying theory [54]–[57]. In contrast to empirical methods to find explanations, ABMs may help understanding a phenomenon by *generating* it [58], [59]. An advantage of models is that distinct factors can be manipulated separately and under controlled conditions, including factors that cannot easily be accessed in real animal groups. ABMs have proven to be well suited to investigate the link between individual behavior and resulting group level patterns in primates [5], [6], [54], [60]–[65] and other species (birds: [66], fish: [67]–[70], insects: [57], [71]–[73]).

The agent-based model we present here is adapted from the DomWorld model of Hemelrijk [5], which in turn was inspired by Hogeweg [74]. The DomWorld model concerns individual variation in dominance rank and dominance-related variation in fleeing frequency. We replicated a simplified version of this model, adding some modifications and extensions. Irrespective of these modifications, our version still exhibits the same characteristics as the DomWorld model. Replicating DomWorld allowed us to compare different models to the DomWorld model, especially with respect to model properties that have not been measured or described previously. In particular, we measured the distribution of encounters among the group members, since the spatial distribution of individuals may affect with whom individuals interact. Our adapted version of DomWorld is, hereafter, referred to as the ‘*fleeing model*’.

We contrasted the *fleeing model* with a model that additionally includes avoidance behavior (*avoidance model*), to investigate how aggressor avoidance, and thus fewer aggressive encounters and less frequently subsequent fleeing, may give rise to different forms of socio-spatial structure. We varied two determinants of avoidance behavior (the rank-difference above which an individual is avoided and the spatial distance within which avoidance is employed). Furthermore, we investigated the isolated effect of avoidance behavior on the socio-spatial structure: by removing individual variation in fleeing frequency from the *avoidance model* we control for any structuring effect that may result from fleeing subsequent to aggressive encounters (*avoidance with fleeing-control model*). An additional goal of this paper is to identify general mechanisms underlying a central-peripheral group pattern. Both the *fleeing model* and the *avoidance model* concern individual variation in movement characteristics, i.e. frequency and direction of fleeing or of spatial avoidance at a distance. In both models subordinates move, i.e. flee or avoid, more frequently than dominants. In a third model (*velocity model*) we test whether individual variation in velocity alone is already sufficient to generate a central-peripheral group pattern.

By investigating how several movement characteristics (fleeing, avoidance and velocity) that may vary across a social group, may result in consistent spatial and encounter structures, a more complete understanding of the emergence of spatial and social group structure and their inter-relatedness is obtained. We present a new, general mechanism and explanation for one of the main questions in primate literature: what causes centrality. More specifically, we investigate the effects of a specific primate behavior: aggressor avoidance at a distance.

## Methods

### The models

Our basic model, the *fleeing model*, is adapted from DomWorld, but differs in the following points. First, we implemented a stable dominance hierarchy (similar to the model in the appendix of [54]). In primates, dominance hierarchies are stable over long periods of time (up to several years, macaques: [75]–[78], gorilla: [79], baboons: [80], [81], capuchins: [82], vervets: [83]) and are altered only incidentally, e.g. after changes in the group composition due to birth, death or migration of individuals [79], [84]. Moreover, we do not aim to study the development of the hierarchy within a group. Instead, we assume that in our group a hierarchy has been already established and does not change over the timeframe of our simulation. Second, we chose to model a larger group than earlier models [5], [6], [54], [64], [85], consisting of 30 instead of 8, 10, 12 or 20 individuals. This more accurately represents group size in many primate species [86], [87] and furthermore results in more informative data regarding the spatial group structure. Third, our adapted grouping procedure allows strayed individuals to find back the group and to move towards it quickly (see section Grouping and movement below). This ensures fast grouping and does not artificially prolong the time spent at the group periphery. Fourth, we restricted the maximum spread of the group to prevent eventual group split-up, while still allowing for flexibility with regard to group spread, individual spatial positions within the group and subgrouping patterns (see section Grouping and movement below). This allowed us to analyze all group members as a single group. Fifth, our model differs from DomWorld in the decision-making procedure subsequent to an encounter. In our model both opponents may decide whether to engage in a fight, as a fight only takes place if both opponents agree to it (see section Social interactions below). This is in contrast with DomWorld where the encountered individual always takes part in the fight, if the encountering agent decided to start a fight [5], [6], [54], [64], [85]. Sixth, we implemented a sigmoid win chance function [88] (as suggested by [89]), instead of a relative win chance function. The latter two adaptations ensured that escalated fights between two individuals distant in rank are rare (see Text S1), as has been suggested by empirical and theoretical work [90]–[92]. Last, in our model, when individuals move within the group they employ a random walk. This contrasts with the DomWorld model, where individuals move straight [5], [6], [54], [85]. In fact, in DomWorld, individuals may only change their heading direction, after an encounter or when other individuals are too far away and need to be approached. An evaluation of the effect of the random movement is discussed below (see Section Robustness of the model).

In the *fleeing model*, individuals behave as follows: (1) Individuals move and orient themselves to have at least three other group members in sight. (2) If this condition is fulfilled, individuals move around randomly. (3) On encounter, individuals may engage in dominance interactions. Each individual's dominance strength determines its ability to win dominance interactions and individuals differ in dominance strength. The loser of a dominance interaction flees from the winner.

To investigate the implications of avoidance behavior for the socio-spatial group structure, we compared a model without avoidance behavior (*fleeing model*) to a model that includes avoidance behavior (*avoidance model*). In the *avoidance model*, individuals follow the same rules as in the *fleeing model*, yet additionally, individuals may avoid potential aggressors at a distance. Thus, the effects of fleeing and avoidance behavior are combined in the *avoidance model*. We also investigated the isolated effect of avoidance by excluding variation in fleeing frequency (*avoidance with fleeing-control model*). Thus, in the *avoidance with fleeing-control model* the structuring effect of fleeing was removed.

To assess whether individual variation in velocity alone may be sufficient to generate a central-peripheral group pattern, we constructed another model (*velocity model*), in which individuals differ merely in their average velocity. In this model, individuals have a tendency to group (rules (1) and (2) above), but there is no variation in fleeing frequency and individuals do not employ avoidance behavior.

Simulations were run using NetLogo 4.0.3 [93]. For our models, we extensively modified an earlier publicly available replication of the DomWorld model by Bryson [54]. The program code of all our models is available for download on our website (http://web.science.uu.nl/behaviour/Evers/index.html). Definitions and values of the model parameters can be found in Table 1. Below, we describe all model procedures in more detail.

**Table 1:** Parameters, definitions and values of the fleeing, avoidance and velocity model.

Parameter	Description	Value
**General parameters**		
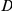	Grid unit	1 m
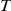	Time step	1 s
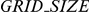	Grid size	300×300 m
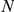	Number of individuals in group	30
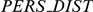	Maximum distance, within which others can be encountered	4 m
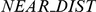	Maximum preferred distance to the group	20 m
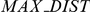	Maximum distance monkeys are able to see	50 m
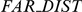	Maximum preferred distance to the furthest group member	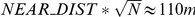
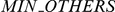	Minimum preferred number of conspecifics within 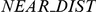	3
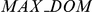	Maximum dominance strength	1.0
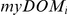	Dominance strength of individual *i*	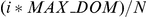
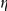	Parameter determining the steepness of the sigmoid function of win chance	6/MAX_DOM
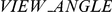	Default view angle	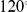
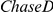	Distance the winner of a fight chases the loser	1 m
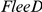	Distance the loser of a fight flees from winner	2 m
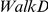	Default distance an individual walks forward	1 m
**Avoidance parameters**		
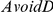	Distance an individual moves away from avoidee	2 m
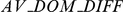	Avoidance dominance difference; difference in strength, above which an agent is considered a potential aggressor and consequently avoided	0.2, 0.4, 0.6
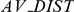	Avoidance distance; spatial distance within which potential aggressors are avoided	5, 15, 25, 35 m
**Velocity parameters**		
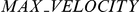	Maximum possible velocity	1, 5, 10, 20, 30 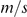
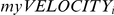	Velocity of individual *i*	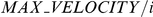

### Environment, initialization and timing regime

The modeled environment is a continuous two-dimensional grid (300×300 grid units) with a torus shape to exclude disturbing border effects. One grid unit is scaled to 1 meter. We chose the size of the grid to be large enough to hold a group with a maximum group spread of around 110 meters (see section Grouping and movement below), while ensuring that real distances between group members were always smaller than distances between group members when measured around the torus. We did not explicitly implement ecological features of the environment; in the model an individual's environment is purely social. This also implies that our model individuals do not engage in foraging behavior. Thus, we model a group that is not traveling.

At the initialization of each simulation run, the x-coordinates and the y-coordinates of all individuals are drawn from a normal distribution around an arbitrarily chosen position on the spatial grid (standard deviation = 10 grid units), independent of their dominance strength. Their initial heading was set to a random number between 1 and 360 degrees.

Our model is event-driven. During a simulation run, individuals' activations are regulated by a timing regime. One time step in the simulation resembles 1 “second”. Agents are activated in a cyclic, asynchronous way. Each time, the agent with the lowest schedule time is activated first. After activation, this agent's next activation is scheduled. The remaining time until its next scheduled activation is randomly drawn from a negative exponential distribution with a mean of 10 time steps:
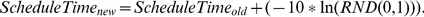
(1)


In other words, events are randomly distributed in time. Scheduled times are on a continuous range. If an action involves other individuals as well, each participant gets scheduled anew for its next action.

### Perception and action-selection

**Figure 1:**
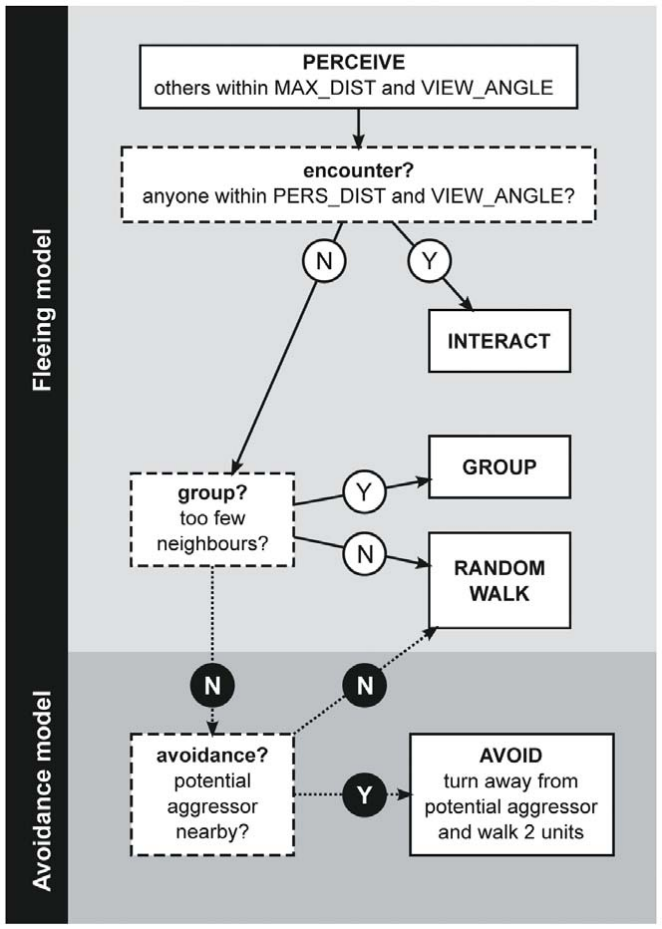
Interaction rules. Model individuals employ a hierarchically organized decision tree. The protocol is starting at the top and resulting in only one of three or four (depending on the model) possible behaviors, depending on the social situation of the individual and the priority of the behaviors. Note that this decision tree does not reflect any temporal order of the behaviors.

On activation, individuals execute an action-selection protocol (Figure 1). This protocol goes through a number of decisions to produce the behavior appropriate to the social situation. The decisions are structured hierarchically according to urgency, e.g. interactions have priority over grouping and grouping has priority over avoidance.

First, individuals check whether other individuals are encountered, i.e. whether other individuals are within a personal distance of 4 m (*PERS_DIST*), which will lead to an interaction (see Interactions section below). If no one is encountered, individuals turn and move towards the group if necessary (see Grouping and Movement section below). In the *avoidance model*, if grouping is not necessary, individuals may further choose to avoid others (see section Avoidance below). If none of the above actions were selected, individuals move randomly within the group (see Grouping and Movement section below).

**Figure 2:**
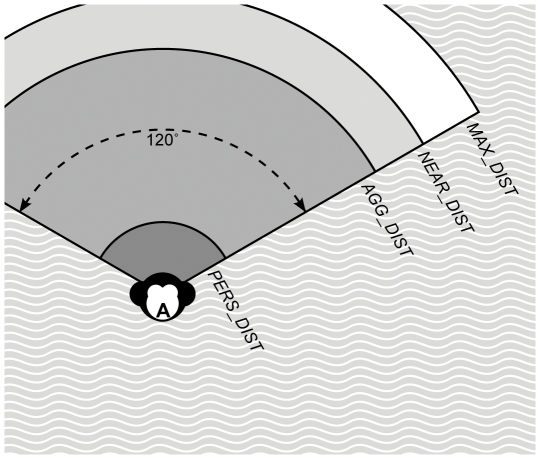
Perception. Model individuals perceive other group members within a default view angle of 120 degrees. The maximum distance within which another can be seen is *MAX_DIST*. Distances in the figure are not to scale.

The identity and spatial position of other group members affects an individual's behavior. Individuals are capable of perceiving the spatial distance and the dominance strength of others that are dwelling within a view angle of 120 degrees and a maximum perceivable distance of 50 m (*VIEW_ANGLE* and *MAX_DIST* in Figure 2). Parameter choices for *PERS_DIST*, *VIEW_ANGLE* and *MAX_DIST* were adapted from (earlier replications of) DomWorld [5], [6], [54], [85].

### Grouping and movement

To stay relatively close to group members, individuals check whether at least three group members (*MIN_OTHERS*) are situated within a distance of 20 m (*NEAR_DIST*) within their view angle (Figure 2). The parameter choice for *MIN_OTHERS* was adapted from van der Post [62], [63], [94]–[96] and the parameter choice for *NEAR_DIST* was adapted from an earlier replication of the DomWorld model [54]. If less than three group members were detected within 20 m, individuals try to find another group member within the maximum distance they can see (50 m), or else within a broader view angle (360 degrees) by looking around (Figure 2). Of the perceived individuals, one is selected randomly and approached by 1 m (*WalkD*). The parameter choice for *WalkD* was adapted from DomWorld [5], [6], [54], [85].

Furthermore, individuals always check the distance towards the furthest group member. If at any time the distance towards the furthest group member exceeds a certain value, *FAR_DIST*, ego will immediately turn towards a randomly selected group member and approach it (for 1 m). *FAR_DIST* depends on the number of individuals in the group (*N*) as follows:
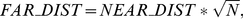
(2)thus, for a group of *N* = 30 individuals, *FAR_DIST* 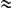
110 m. The parameter value for *FAR_DIST* was chosen arbitrarily. Note that by approaching a random group member, the probability of selecting another individual that just walked away from the group itself is small. This grouping procedure ensures that all group members remain within a certain distance from each other. Subgroups may form, but eventual group split-up is prevented in our model.

Movement of the model individuals is either motivated by explicit social factors, such as grouping, fleeing, chasing or avoidance, or is else implemented as a random walk. When executing a random walk, individuals simply move forward (for 1 m) and with a chance of 0.5, they then turn randomly up to 180 degrees to the right or left.

### Social interactions

When other group members are perceived within a personal distance of 4 m, ego chooses the nearest individual as an interaction partner. In our models interactions are always dyadic. For each interaction, the partner choice is recorded and scored as an encounter. Thus, encounters are directed from one individual (ego, who perceived the other first) to another (chosen partner).

**Figure 3:**
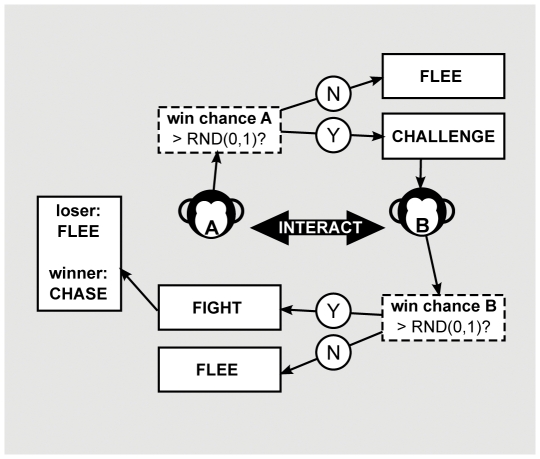
Interaction rules upon encounter. Upon encounter, an agent may either challenge or flee from the opponent. After being challenged, the opponent may either flee or agree to a fight.

On encounter, ego may either challenge its interaction partner or flee from it for 2 m (Figure 3). This decision depends on the chance of winning a fight with the opponent. This win chance is dependent on both opponents' dominance strength. When initializing a simulation run, each model individual gets assigned a fixed value for its dominance strength (*myDOM*), ranging from 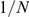 (for the lowest-ranking individual) to 1.0 (highest-ranking). Note that this choice of scaling the dominance values between 0 and 1 is arbitrary and does not affect our results. The chance of individual *A* winning against individual *B*, 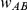, is then calculated as:
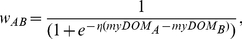
(3)where the parameter 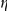 describes the steepness of the sigmoid win chance function and 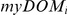 is the dominance strength of individual *i*. Note that by definition the win chances of both opponents add up to 1 and the opponents win chance is thus: 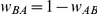. A higher difference in dominance strengths results in a higher chance of winning the fight for the dominant individual. Note that the win chance 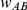 in our model is the same whenever the difference in dominance strengths, 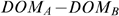, is the same [89]. Note further that we chose 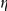 such that the distribution of win chances among the group members is comparable to the DomWorld model, with win chances ranging from 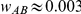 (for 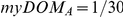 and 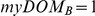) to 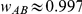 (for 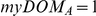 and 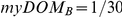).

On encounter, ego challenges its opponent when its expected win chance is higher than a randomly drawn number between zero and one:
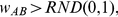
(4)and flees when it is lower. As a response to a challenge, the opponent may either reject or agree to engage in a fight, depending on its own expected win chance (Figure 3). If the opponent's win chance 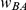 is higher than a new randomly drawn number, a fight will start, otherwise the individual declines and flees. As soon as one of the two interacting individuals declines and subsequently flees, the conflict is settled and no fight takes place.

If no fight took place, the fleeing individual turns away from its opponent. With a chance of 0.5, its opponent visually orients towards a random direction. Staying oriented towards the opponent would result in repeated interactions. However, since the fleeing individual acknowledged its opponent's higher status, the opponent can orient elsewhere.

In our models, only if both individuals agree to a fight does an actual fight take place (Figure 3). The winner of a fight is stochastically determined: individual *A* wins from *B*, when its win chance, 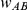, is higher than a new randomly drawn number between zero and one (Equation 3 and 4).

After a fight, the loser flees from the winner for 2 m (*FleeD*), while the winner chases the loser by running after him for 1 m (*ChaseD*) (Figure 3). Parameter choices for *FleeD* and *ChaseD* were adapted from DomWorld [5], [6], [54], [85]. Bryson [54] proposed to reinterpret the fight and subsequent fleeing behavior in the DomWorld model as displacement behavior. Our model implements an even richer behavioral differentiation. When individual *A* perceives *B* nearby and immediately flees, we may call this unprovoked fleeing. When individual *A* flees only after *B* signaled its fighting intention, we may call this fleeing after threat. When both individuals signaled their fighting intention, a real fight takes place.

In contrast to the DomWorld model, we did not implement so-called “wiggling” in which the winner turns about a certain angle after chasing the loser. This was implemented in the DomWorld model to (artificially) prevent too many repeated interactions between the same individuals [5], [97]. However, we found that this work-around can affect the socio-spatial structure. For a short analysis of the effect of “wiggling” see the Text S2.

Outcomes of fights have no further implications for the behavior or for the dominance strength of the participants. Thus, we model a primate group with a stable dominance hierarchy where dominance strength is not updated after a fight (like the model in the Appendix of Bryson *et al.* [54]; but unlike the DomWorld model of Hemelrijk [5]).

The interaction procedure described above results in low-ranking individuals losing and fleeing more often than high-ranking ones. In the *avoidance with fleeing-control model* and in the *velocity model* we controlled for individual differences in fleeing rate by simply assigning a win chance of 0.5 to each individual, independent of its actual dominance strength. In this way, fleeing rates were equal among individuals, while other properties, such as avoidance behavior or velocity, did still differ.

### Avoidance

In addition to all procedures described above, individuals in the *avoidance model* may avoid known potential aggressors at a distance. This contrasts with earlier models where individuals may flee from a dominant only directly after encountering it [5], [6], [54], [64], [85]. How and to what extent avoidance behavior is employed is determined by two parameters: *AV_DOM_DIFF* and *AV_DIST*, which will be explained below.

Whether an individual identifies another as a potential aggressor depends on the difference of both individuals' dominance strength. The parameter *AV_DOM_DIFF* (avoidance dominance difference) describes the minimum difference in dominance strength between two individuals that elicits avoidance behavior in the subordinate. Thus, individual *A* avoids individual *B*, if:
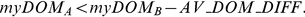
(5)


Values for *AV_DOM_DIFF* may be varied from zero to one. A high value for *AV_DOM_DIFF* represents a system where only the lowest-ranking subordinates avoid only a few highest-ranking individuals. A low value for *AV_DOM_DIFF* means that subordinates avoid most higher-ranking individuals, mimicking a more despotic group with more pronounced aggression and frequently employed avoidance.

Whether ego avoids a detected potential aggressor is dependent on the spatial distance to that animal. The parameter *AV_DIST* (avoidance distance) describes the spatial distance within which subordinates avoid aggressors (Figure 2); ego avoids potential aggressors that are perceived at a distance smaller than *AV_DIST*. Values for *AV_DIST* may be varied from zero to *MAX_DIST* (Table 1). A low *AV_DIST* value would result in subordinates avoiding only those potential aggressors that were already very close. A high value for *AV_DIST* would result in subordinates avoiding also those potential aggressors that were still at a large distance, mimicking a more despotic group with more pronounced aggression and frequently employed avoidance. Therefore, the number and the identity of potential aggressors may differ among individuals, depending on their dominance strength and their spatial position within the group.

The actual avoidance behavior of detected potential aggressors is implemented in the following way: Reacting on another individual directly after encounter, i.e. after perceiving any other individual within *PERS_DIST* still has the highest priority. If no encounter took place, ego checks whether there are too few neighbors perceived within *NEAR_DIST*, which would result in grouping behavior. When no encounter takes place and no grouping behavior is necessary, ego checks whether there is a potential aggressor within *AV_DIST* (Figure 1). If potential aggressors are detected, the nearest one is selected and avoided: ego turns away from this individual and walks away for 2 m.

### Velocity

In the fleeing model and the avoidance model, subordinates flee from or avoid dominants. This suggests that on average subordinates move over larger distances, compared to dominants. To check, whether this variation in the amount of movement may be sufficient to generate a central-peripheral group pattern we developed the velocity model, where individuals merely differ in their average velocity, i.e. the average distance they walk per time interval (in meters/second). In this model, we made velocity directly dependent on dominance strength, thus subordinates walk greater distances in the same time interval compared to dominants. Velocity of individual *i* is calculated as follows:
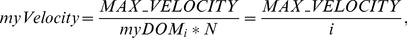
(6)where, 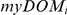 is the dominance strength of individual *i*, *N* is the total number of individuals within the group and *MAX_VELOCITY* is the maximum possible velocity within the group. Note that in the *velocity model*, individuals do not differ in their fleeing rates nor do they employ any avoidance behavior (see section Social interactions above).

### Data collection and parameter settings

To assess socio-spatial group properties within each model, we used several measures. To measure how individual differences in fleeing frequency, avoidance tendency and velocity were related to the individuals' spatial position within the group, we calculated each individual's distance to the arithmetic center of the group. The coordinates of the arithmetic center of the group were calculated as follows:
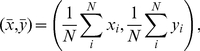
(7)where *N* is the number of individuals in the group and 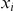 and 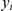 are the spatial coordinates of individual *i*. When the group was wrapped around a border of the field in the direction of the x or y-axis, the respective coordinates (*x* or *y*) of the individuals at the low end of the field were increased by the length of the field in the respective direction for the calculation. We also calculated centrality-peripherality using circular statistics and the mean spatial direction of all others around an individual. This procedure is described and discussed elsewhere (see 98–100 and Figure 4a in [6]). Values for centrality-peripherality are similar to the distances to the arithmetic center of the group, except that the centrality-peripherality measure is normalized and scaled to values between 0 and 1. Here, we only discuss the results for the distances to the arithmetic center of the group, as they are more informative considering the group spread and as group size is the same in all the models presented here.

**Figure 4:**
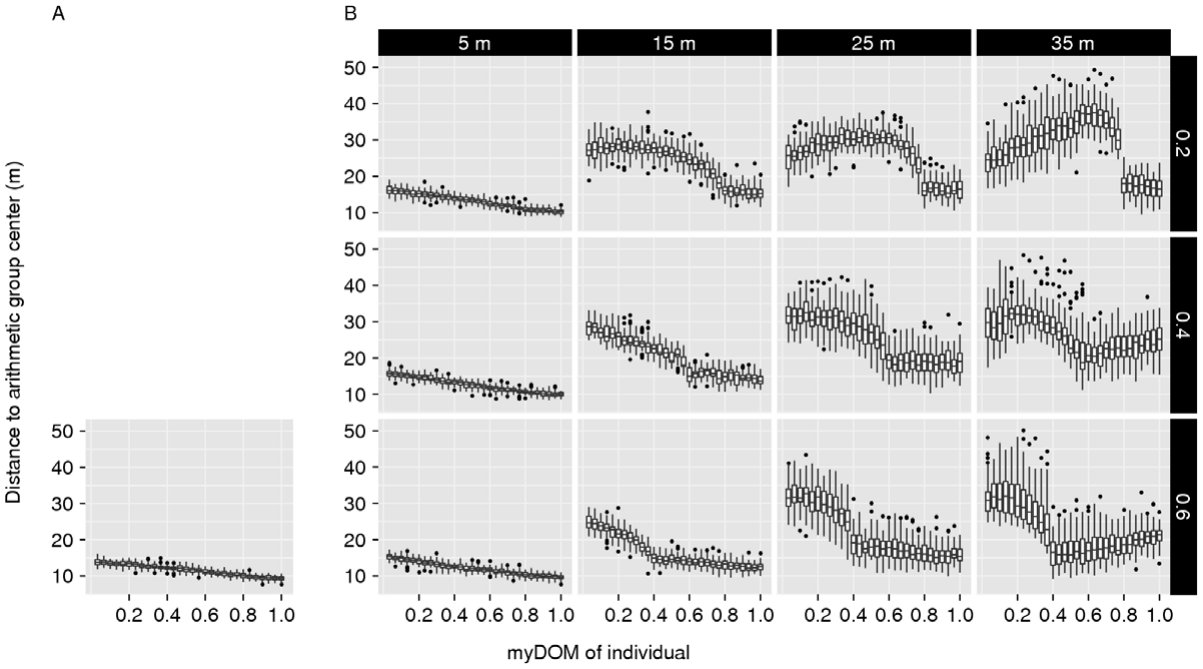
Centrality of dominants. This figure shows the relationship between an individual's dominance strength (myDOM) and its centrality (distance to the arithmetic center of the group in meters) for different models. (A) *Fleeing model*. (B) *Avoidance model* (with different combinations of *AV_DOM_DIFF* (vertically, 0.2–0.4) and *AV_DIST* (horizontally, 5–35 m). Small distances to the arithmetic group center indicate more central positions. When the relation between dominance strength and centrality is steeper, centrality of dominants is more pronounced. Depending on the model, a low myDOM further implies low win chance and thus frequently employed fleeing behavior (*fleeing* and *avoidance model*) and frequently employed avoidance behavior (*avoidance model*). Boxplots show values of 50 simulation runs, averaged over time.

We measured differences in spatial group spread by recording the furthest neighbor distance within the group (the distance between the two individuals in the group that are furthest away from each other).

We assessed how dyadic distances, as well as the number of encounters were distributed among all possible dyads. The spatial dyadic distances were simply recorded over time. To measure the total number and direction of encounters, i.e. the encounter structure, we recorded the identity of those group members that ego had selected as opponents.

A single simulation was run for 72,000 time steps, which resembled 20 observation “hours”. We recorded data during the last 10 “hours”, to avoid transient spatial and social group effects due to the initial random placement. This time period is sufficiently long to measure patterns emerging from the short-term scale (inter)actions in the model. All measures of the socio-spatial group structure (distance to arithmetic center of the group, centrality-peripherality, spatial group spread, dyadic distances and encounter structure) were recorded every 900 time steps which was equivalent to 15 “minutes”. All measures, except the number of encounters per dyad, were averaged over recorded time for each simulation run. For the number of encounters per dyad all occurrences were recorded. For each model 50 independent simulations were run per parameter setting.

In the *avoidance model*, the parameter *AV_DOM_DIFF* was varied between 0.2 and 0.6, and the parameter *AV_DIST* was varied between 5 m and 35 m. In the *velocity model*, the parameter *MAX_VELOCITY* was varied between 1 m/s and 30 m/s. See Table 1 for an overview of all parameters used in our models.

### Experimental set-up

First, we confirmed whether the properties of our *fleeing model* were similar to earlier results published on the DomWorld model. To then assess the effect of avoidance behavior on socio-spatial group properties, we contrasted the *fleeing model* to the *avoidance model*. The *fleeing model*, where individuals do not employ any avoidance behavior, would correspond to groups where avoidance might simply not be necessary, e.g. due to very low levels of aggression. In contrast to that, the *avoidance model* reflects a whole range from little to intensive avoidance behavior (depending on the parameter settings), which would correspond to groups ranging from low-level to severe aggression. To investigate, whether the socio-spatial group properties that emerged in the *avoidance model* depend on the more frequent fleeing of subordinates, we implemented the *avoidance with fleeing-control model*. In this model we controlled for variation in fleeing frequency to measure the isolated effect of avoidance. Thus, in the *avoidance with fleeing-control model*, subordinates flee equally often as dominants after a fight. This model does not attempt to represent real primate groups, but it allows us to disentangle several factors and their effects, which are usually interconnected in the real system. Finally, we measured group level properties of the *velocity model*, in which individuals merely vary in their velocity. In other words no avoidance is employed and subordinates flee equally often as dominants. The *velocity model* does also not attempt to represent real primate groups. Rather, it allows us to investigate, whether variation in velocity alone may be already sufficient to generate a central-peripheral pattern in groups of entities. The experimental set-up is summarized in Table 2.

**Table 2:** Experimental set-up and characteristics of the compared models.

	Factor:	Fleeing frequency	Avoidance tendency	Velocity
**1.**	Fleeing model	**Variable**	Not employed	Equal
**2a.**	Avoidance model	Variable	**Variable**	Equal
**2b.**	Avoidance fleeing-control model	Equal	**Variable**	Equal
**3.**	Velocity model	Equal	Not employed	**Variable**

## Results

### Fleeing model

**Figure 5:**
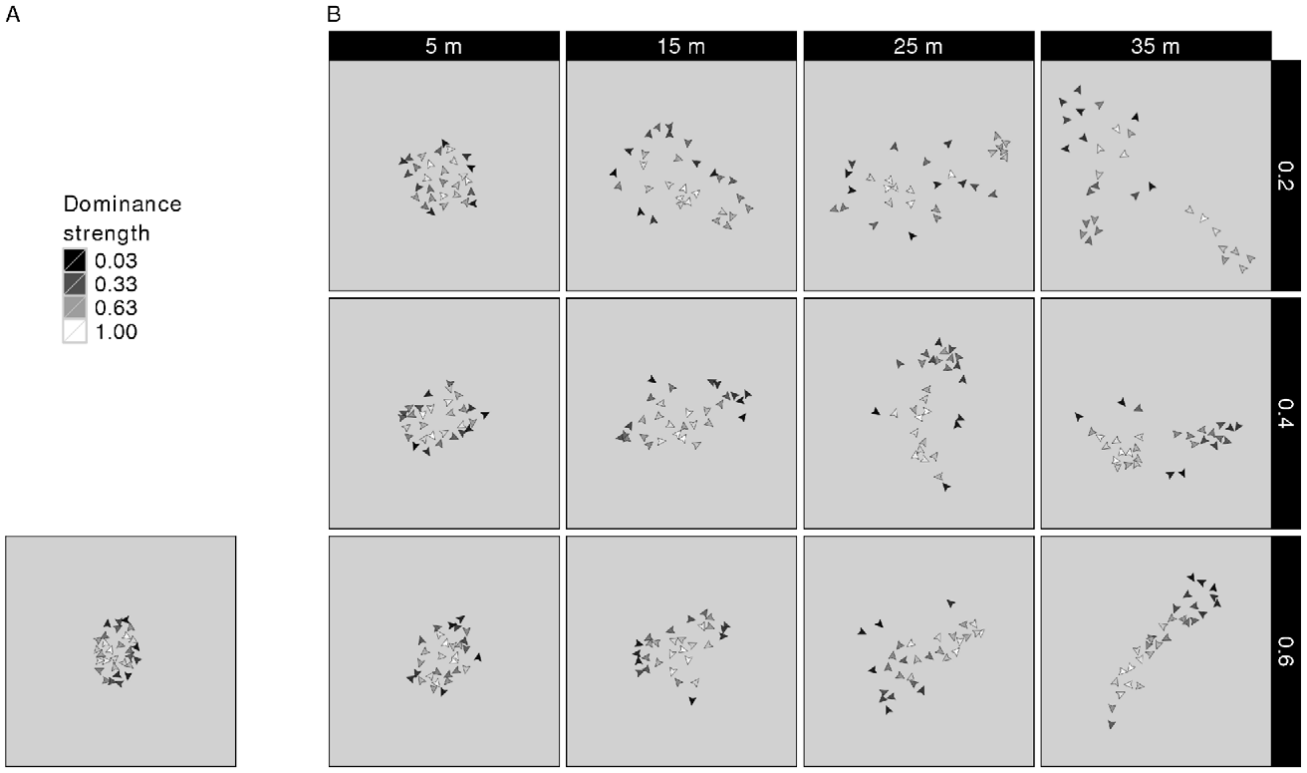
Snapshots of the socio-spatial group structure. This figure shows snapshots of the spatial composition of the group members for different models. (A) *Fleeing model*. (B) *Avoidance model* (with different combinations of *AV_DOM_DIFF* (vertically, 0.2–0.4) and *AV_DIST* (horizontally, 5–35 m). Shown is a 100 by 100 meters excerpt of the total grid at one arbitrary point in time. Each arrowhead represents an individual. White shade represents a high dominance strength, dark shade represents a low dominance strength. The heading of an arrowhead represents the individual's visual orientation. For further implications of an individual's dominance strength depending on the model, see the Figure 4 legend.

As described above, the *fleeing model* is a modified version of DomWorld model [5]. In this *fleeing model*, a (weak) central-peripheral group structure arose with dominants more often at the group center and subordinates more often at the periphery (Figure 4A). The lower an individual's dominance strength, the more likely it was to lose a dominance interaction. Thus, subordinates fled more often just before or after a fight, thereby moving away from higher-ranking individuals. Since the most dominant animal (alpha animal) was the individual that most others usually fled from, the alpha was automatically found at a spatially central position in the group. Since subordinates fled from most other group members, they were found more often at the periphery of the group (see Figure 5A for an example snapshot). This self-sorting of the model individuals, according to their dominance strength, arose through self-organization. As a result distance from the group center, correlated with dominance strength (Figure 4A).

**Figure 6:**
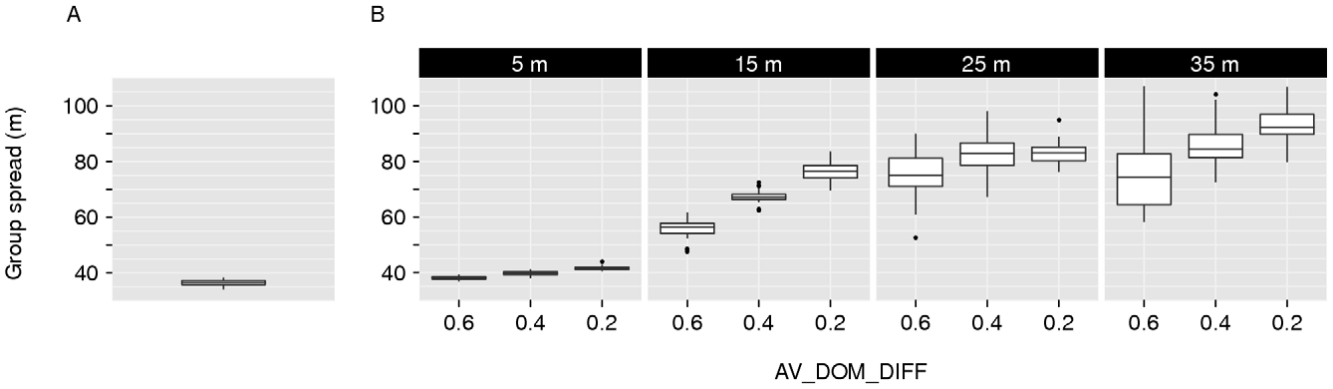
Group spread. This figure shows the group spread (in meters) for different models. (A) *Fleeing model*. (B) *Avoidance model* (with different combinations of *AV_DOM_DIFF* (x-axis, 0.2–0.4) and *AV_DIST* (horizontally, 5–35 m). Boxplots show values of 50 simulation runs, averaged over time.

**Figure 7:**
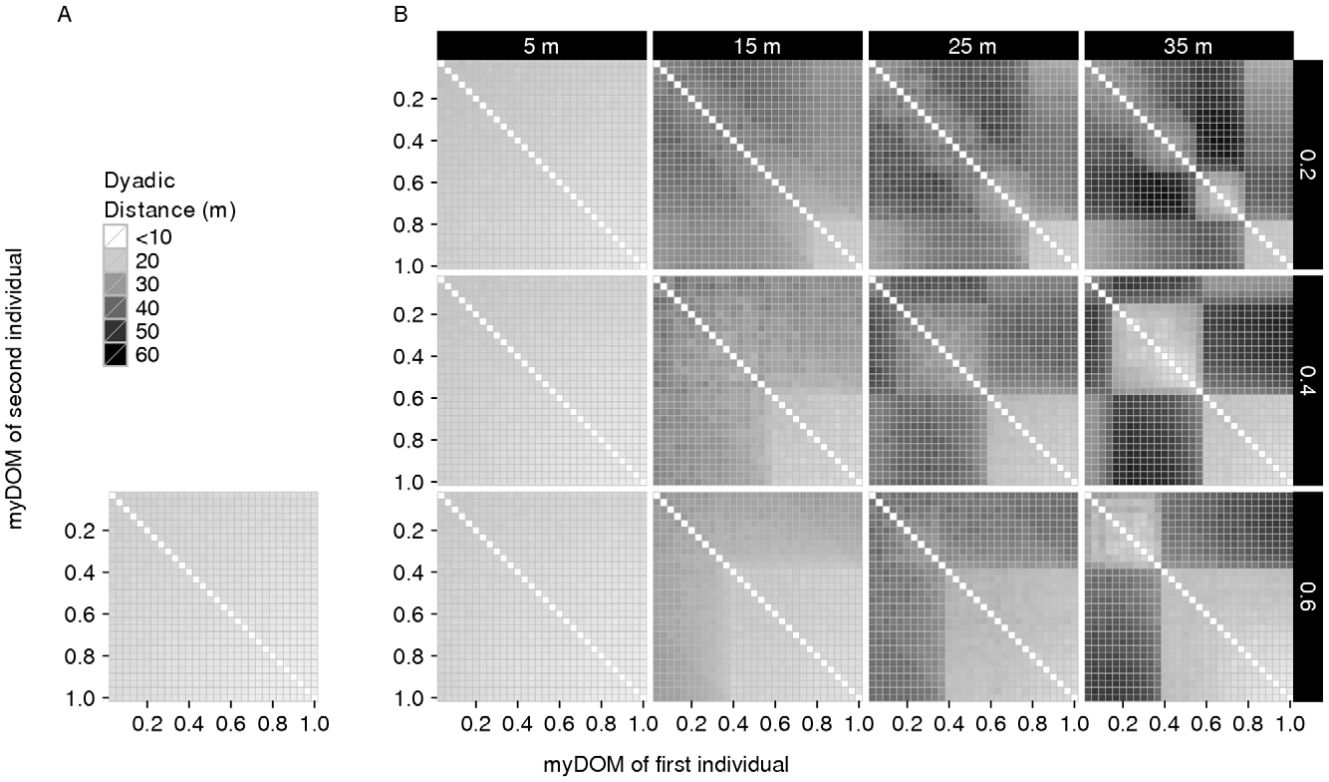
Spatial structure. This figure shows the distribution of dyadic distances (in meters) among the individuals of a group for different models. (A) *Fleeing model*. (B) *Avoidance model* (with different combinations of *AV_DOM_DIFF* (vertically, 0.2–0.4) and *AV_DIST* (horizontally, 5–35 m). The x-axis shows the dominance strength (myDOM) of the first individual and the y-axis the dominance strength of the second individual per dyad. For further implications of an individual's dominance strength depending on the model, see the Figure 4 legend. Plots show the mean values of 50 simulation runs, averaged over time. Darker shades represent larger dyadic distances. Values at the diagonal are by default not applicable. Note that the distance matrices are by definition symmetrical.

**Figure 8:**
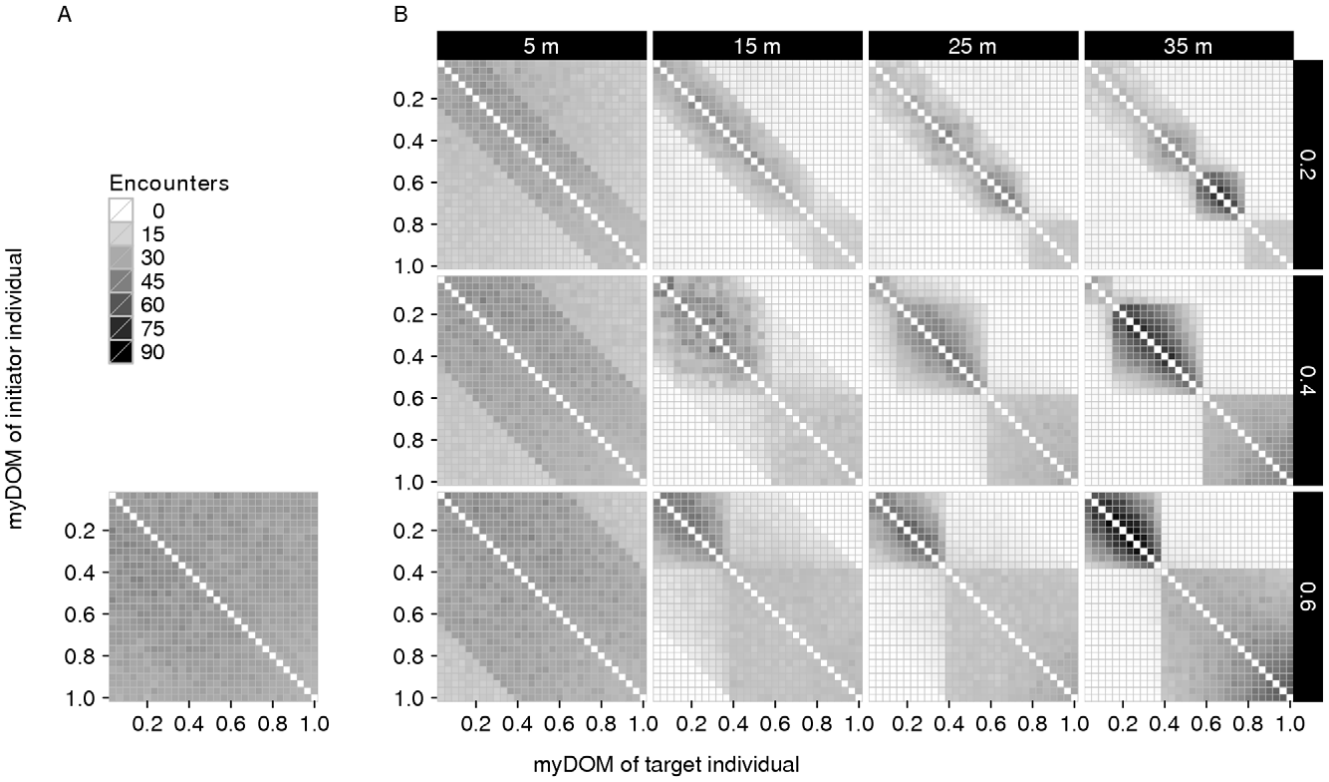
Encounter structure. This figure shows the distribution and direction of encounters among the individuals of a group for different models. (A) *Fleeing model*. (B) *Avoidance model* (with different combinations of *AV_DOM_DIFF* (vertically, 0.2–0.4) and *AV_DIST* (horizontally, 5–35 m). Encounters are directed from initiators (y-axis) to targets (x-axis), both are ordered by dominance strength (myDOM). For further implications of an individual's dominance strength depending on the model, see the Figure 4 legend. Plots show the mean values of 50 simulation runs. Dark shades represent frequent encounters. Values at the diagonal are by default not applicable.

For comparison with the other models in this paper, the following results of the *fleeing model* are important. The repulsive force of fleeing counteracted the attractive force of grouping and affected how much the group was spread out and how it was patterned in space. The spread of the group in the *fleeing model* was small (Figure 6A): the average furthest neighbor distance was 36.4
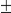
1.0 m (mean 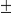 standard deviation, N = 50 simulation runs). Consequently, the average dyadic distances were similarly small among all individuals (Figure 7A). Following from this, the frequency and direction of encounters were similar among all individuals, i.e. the encounter structure was not differentiated (Figure 8A).

### Avoidance model: Spatial structure

To assess the effect of avoidance behavior on socio-spatial group properties, we compared the *fleeing model* to the *avoidance model*, while varying values for the distance within which an individual avoided potential aggressors (*AV_DIST*, varied from 5 m to 35 m) and the difference in dominance strength above which avoidance was employed by the subordinate (*AV_DOM_DIFF*, varied from 0.2 to 0.6). Just as in the *fleeing model*, we observed a central-peripheral distribution of animals, sorted according to their dominance rank (Figure 4B). When avoidance was employed at a small distance (*AV_DIST* = 5 m), the central-peripheral group structure was comparable to the *fleeing model*. Because avoidance was only employed when a potential aggressor was very close the behavioral and spatial consequences were comparable to fleeing from an opponent after encountering it within *NEAR_DIST* (4 m). When avoidance was employed at intermediate distances (*AV_DIST* = 15–25 m), the socio-spatial structure became more pronounced (Figure 4B); individual distances to the group center were more differentiated than in the *fleeing model*. This is reflected in the steeper slope in Figure 4B as compared to the slope in Figure 4A (the *fleeing model*). The spatial group structure in the *avoidance model* is illustrated in some example snapshots in Figure 5B. Note that in the *avoidance model*, individuals with 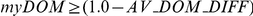 did not avoid any other individuals by definition (see Equation 5). These dominant individuals formed a subgroup at the center of the group. This central subgroup showed small variation in average spatial distance to the group center (Figure 4B). In fact the central subgroup in the *avoidance model* behaved just like individuals in the *fleeing model*, as no avoidance was employed by these individuals and only variation in fleeing frequency structured the spatial properties within this subgroup. When avoidance was employed at very large distances (*AV_DIST* = 35 m) the central-peripheral group structure broke down (see subsection Subgroup formation below).

Groups in the *avoidance model* were more spread out compared to the *fleeing model* (Figure 6B). The furthest neighbor distance was ranging from 38.0
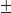
0.6 m (for *AV_DOM_DIFF* = 0.6 and *AV_DIST* = 5 m) to 92.6
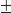
6.0 m (for *AV_DOM_DIFF* = 0.2 and *AV_DISTAFF* = 35 m, mean 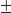 standard deviation, N = 50 simulation runs). Higher values for *AV_DIST* resulted in larger group spread, as potential aggressors were avoided at larger distances. Lower values for *AV_DOM_DIFF* yielded a larger group spread, as more group members needed to be avoided (Figure 6B).

To assess the isolated effect of avoidance behavior on socio-spatial group patterns, we measured the relationship between spatial distance to the group center and dominance strength in the *avoidance with fleeing-control model* (a model without individual variation in fleeing frequency). In the *avoidance with fleeing-control model*, a similar spatial structure to that of the *avoidance model* emerged (Figure S1), although groups were less spread out for avoidance at small and intermediate distances. The furthest neighbor distance was ranging from 30.7
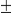
0.4 m (for *AV_DOM_DOM_DIFF* = 0.6 and *AV_DIST* = 5 m) to 93.3
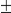
9.5 m (for *AV_DOM_DOM_DIFF* = 0.2 and *AV_DIST* = 35 m, mean 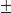 standard deviation, N = 50 simulation runs). Furthermore, the variation in distance to the group center among the central dominants disappeared (Figure S1). Because we controlled for variation in fleeing, these central individuals were in fact identical to each other and only differed in the degree to which they were avoided by others.

### Avoidance model: Subgroup formation

When avoidance was employed at large distances (*AV_DIST* = 35 m), dyadic distances between avoiders and avoidees became larger. Eventual splitting-up of the group was restricted in our model (see Methods), but individuals formed subgroups that were spatially separated. Subgroups emerged, consisting of individuals of similar rank that did not avoid each other (Figure 7B). As a result of this spatial structure, almost no encounters took place between individuals from different subgroups (Figure 8B). The number and size of subgroups depended on *AV_DOM_DIFF*, with more and smaller subgroups for low values of *AV_DOM_DIFF*. For example, if *AV_DOM_DIFF* = 0.2, individuals with dominance strength higher than 0.8 formed the alpha subgroup, individuals with dominance strength between 0.6 and 0.8 formed the beta subgroup, and so on (Figure 7B and 8B).

In addition, for avoidance at large distances (*AV_DIST* = 35 m), the general central-peripheral group pattern broke down (Figure 4B). When avoidance at large distances (*AV_DIST* = 35 m) was combined with low *AV_DOM_DIFF* (0.2), lower-ranking individuals formed several subgroups (e.g. beta, gamma and delta) around the central alpha subgroup, arranged spatially according to average subgroup rank. However, the probability that a low-ranking subgroup was “driven apart” by an approaching, more dominant subgroup increased with decreasing average subgroup rank. Therefore, the lowest-ranking individuals could not aggregate as a subgroup, as they were constantly forced to avoid approaching potential aggressors. While the beta subgroup could keep a safe distance from the alpha subgroup, the still lower-ranking subgroups (e.g. gamma and delta) were forced to occupy spatial positions closer to the center, in-between the central alpha subgroup and the peripheral beta subgroup (for an example snapshot of the spatial configuration at this parameter setting see Figure 5B). As the maximum group spread was restricted in the model, these lower-ranking subgroups could not aggregate at an even greater distance from the higher-ranking subgroups. Without this restriction, avoidance at a large distance would have caused the group to split-up into separate subgroups (see section Robustness of the model).

When avoidance at large distances (*AV_DIST* = 35 m) was combined with high *AV_DOM_DIFF* (0.6), the spatial patterning of the subgroups was rather different than for lower *AV_DOM_DIFF*. Individuals with dominance strength lower than 0.4 avoided others with a dominance strength higher than 0.6. Thus, individuals of intermediate dominance strength did not avoid others and were also not avoided by others, but acted as a spatial buffer. Individuals sorted themselves according to their dominance rank resulting in spatially central intermediate-ranking individuals, with the alpha subgroup (the avoided individuals) on one side, and the low-ranking subgroup (avoiders) on the other side (for an example snapshot of the spatial configuration at this parameter setting see Figure 5B).

In sum, when avoidance was employed at large distances, individuals of similar rank formed subgroups, which was reflected in the spatial and encounter structure.

### Avoidance model: Encounter structure

In the *avoidance model*, the average distances were similar among all individuals for avoidance at small distances (*AV_DIST* = 5 m) and comparable to the *fleeing model* (Figure 7B). However, encounters happened more often between individuals of similar rank than between individuals of distant rank (darker band around the diagonal in Figure 8B). Note that the range of “preferred” dominance values of interaction partners (the width of the band around the diagonal in Figure 8B) is determined by the parameter *AV_DOM_DIFF* (Figure 8B): a lower *AV_DOM_DIFF* allows interactions only between individuals very close in rank. While individuals of too high a rank would be avoided by a specific individual, individuals of too low a rank would themselves avoid this specific individual.

When *AV_DIST* was increased to 15 m, frequent avoidees (high-ranking individuals that were avoided) at the center of the group were surrounded by frequent avoiders (low-ranking individuals) at the periphery (Figure 8B). While the individuals still formed a coherent group spatially (see Figure 5 for an example snapshot of the spatial configuration), encounters were not only restricted to similar-ranking individuals, but also to either avoidees or avoiders (Figure 8B). Avoidees were individuals that employed no avoidance behavior at all (as for these individuals there were simply no potential aggressors to be avoided), while avoiders were avoiding at least one individual from the group of avoidees, thereby avoiding the whole group of avoidees (given a large enough *AV_DIST*).

When avoidance was employed at very large distances, subgroups formed, consisting of avoiders and avoidees (see subsection Subgroup formation above). These subgroups were spatially distinct and encounters were now restricted to individuals from the same subgroup (Figure 8B).

### Velocity model

**Figure 9:**
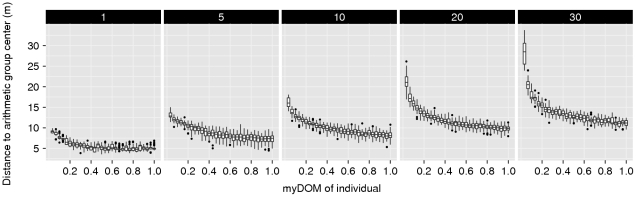
Centrality of dominants in the *velocity model*. This graph shows the relationship between an individual's dominance strength (myDOM) and its centrality (distance to the arithmetic center of the group in meters) for the *velocity model* for a range of values for *MAX_VELOCITY* (horizontally, 1–30 m/s). Small distances to the arithmetic group center indicate more central positions. When the relation between dominance strength and centrality is steeper, centrality of dominants is more pronounced. In this model an individual's velocity has been made inversely related to myDOM. Boxplots show values of 50 simulation runs, averaged over time.

In the very simple *velocity model*, which did not include avoidance behavior and in which there was no variation in fleeing frequency, also a central-peripheral pattern emerged: Individuals were sorted in space according to their average velocity, with fastest moving individuals at the periphery (Figure 9; note that in this model an individual's velocity was made inversely related to myDOM). In the *velocity model* the spread of the group was dependent on the maximum possible velocity. With a higher maximum velocity, a larger group spread (see Figure S2) and a more pronounced central-peripheral group structure emerged (see Figure 9).

### Robustness of the model

We conducted a number of control experiments in our model, to check for implementation-based biases.

First, to ensure that our results do not depend on the specific timing regime chosen in our model, we implemented a model with a different timing regime. Here, an individual's next schedule time was a continuous random number between 0 and 20, thus also with a mean of 10 time steps. This change of the timing regime yielded the same patterns as our original model (data not shown).

Second, to check whether our results would also apply for smaller groups, we ran the model for group size *N* = 10. This model yielded similar result as the original model. However, we observed no subgroup formation when aggressors were avoided at large distances. This was due to the limited number of individuals. With only a few (“preferred”) group members nearby, individuals move towards others rather than avoiding potential aggressors (data not shown).

Third, we checked whether our results were affected by the degree of randomness in the random walk procedure. This degree of randomness is determined by the random angle individuals can turn about. With a lower turning angle, individuals move more persistently in a particular direction. The movement direction of individuals is probably highly persistent when foraging, but much less persistent when individuals are not traveling. In our original model individuals could turn up to 180 degrees to the left or right. When we ran our model with a maximum turning angle of 45, 90 and 135 degrees, we obtained similar results to the original model. However, a lower maximum turning angle, and thus more persistent movement, caused a larger group spread (see Figure S3 for the group spread in the *fleeing model* with different maximum turning angle). Note, that this is similar to the results obtained from the *velocity model*; if all individuals move faster (or more persistently in a particular direction) all individuals end up further away from the group center. As a result of the larger group spread, the number of encounters between individuals decreased. This in turn decreased the structuring effect of fleeing (after encounter). Therefore, when we ran the *avoidance model* with a lower maximum turning angle (45 degrees), we could still observe the spatial patterns that resulted from avoidance behavior (central-peripheral group structure or subgroups), while the spatial structuring among avoidees (which came about only due to fleeing) disappeared (see Figure S4).

Fourth, we were interested to which degree our subgrouping patterns depended on the discrete cut-off chosen in the behavioral rules for aggressor avoidance. In our model, individuals always avoided others, when their difference in dominance strength was larger than a certain value. To test how much our results depended on this assumption, we also implemented a more probabilistic way of avoidance behavior, where higher-ranking individuals were avoided according to an avoidance chance. This avoidance chance was implemented as a sigmoid function, which is characterized by its inflection point and the slope at the inflection point. For the inflection point we chose the same values as for *AV_DOM_DIFF* in the original model (namely 0.2, 0.4 and 0.6). Around these values (for difference in dominance strength) the chance of avoiding the particular individual changes (more or less rapidly, depending on the slope) from zero to one. For the slope at the inflection point we tested the values 5, 15, 30, 60 and 120. Note, that the discrete cut-off in our original model could be approximated by this sigmoid function with an infinite slope. The *avoidance model* with probabilistic avoidance behavior obtained results similar to the original model, whenever the slope of the avoidance chance function was steep enough (see Figure S5). Note, that for subgroup formation we needed larger avoidance distances than in the original model (see Figure S6), because with probabilistic avoidance chance, avoidance is less strict and thus less often employed. When the slope was very low (slope = 5), no subgroup formation was observed (see Figure S7). With a low slope of the avoidance chance function, individuals avoided all higher-ranking individuals with a certain probability, therefore no individuals were left to form a subgroup with. Moreover, the inflection point of the avoidance chance function had two opposing effects on subgroup formation. A very low value for the inflection point (0.2) resulted in individuals avoiding most higher-ranking individuals, while having just a few potential partners to form a subgroup with. On the other hand, a high value for the inflection point (0.6) resulted in individuals avoiding only very few higher-ranking individuals, while having many potential partners to form a subgroup with. Therefore, the most pronounced subgroup formation occurred at intermediate values for the inflection point (0.4) (see Figure S5). From this we can derive the following conditions for subgroup formation: a sufficient number of individuals should not avoid each other, allowing the formation of a subgroup, while a sufficient number of other individuals (and their subgroup) should be avoided at a sufficiently large distance.

Fifth, we tested whether switching off the restriction of the maximum group spread would result in spatially separated subgroups. As expected, in the *avoidance model* with large enough *AV_DIST* the group split up in separate subgroups, which tended to move away from each other (see Figure S8 for some example snapshots of the spatial configuration).

Last, we tested the effect of *FleeD* on the spatial group structure, in particular the group spread. We tested a range of values for *FleeD* (1 m, 2 m, 5 m, 10 m, 20 m) in the *fleeing model*. As expected, larger *FleeD* resulted in groups that were more spread out (see Figure S9). This is again in line with the results obtained from the *velocity model*, if individuals move faster, they end up further away from the group center, thus the group is more spread out. As fleeing was mostly employed by lower-ranking individuals, and almost never by high-ranking individuals, the socio-spatial group structure became more differentiated (see Figure S10). The larger group spread resulted in larger dyadic distances. Similar to the original *fleeing model*, the encounter structure was not differentiated, because the spatial distances were similar among all individuals (see Figure S11).

## Discussion

### Emergence of central-peripheral spatial group structure

We identified three different factors that may drive the emergence of a central-peripheral spatial structure in primates, and possibly other group-living species as well: individual variation in fleeing frequency, in avoidance behavior or in velocity.

In the *fleeing model*, the resulting spatial group structure was consistent with earlier findings [5]: the fleeing behavior of subordinates shaped a central-peripheral structure. In line with other model-based research ([64] and the model in the Appendix of Bryson *et al.* [54]), the emergent spatial structure did not depend on winner-loser effects, but arose also with a stable dominance hierarchy. Similar to the *fleeing model*, avoidance of potential aggressors at small or intermediate distances (*avoidance model*) resulted in a central-peripheral group structure with avoiders at the periphery and avoidees at the center, though in the *avoidance model* this spatial structure was more pronounced. Moreover, when we controlled for individual variation in fleeing frequency (by keeping win chances equal for all individuals), a similar spatial structure to that in the *fleeing* and in the *avoidance model* emerged. The self-organizing principle here is analogous to the effect of fleeing: subordinates avoid mostly dominants, which in turn remain at the center of the group. However, in contrast to fleeing upon an aggressive encounter, avoidance already operates at a distance. In the third model (the *velocity model*), we showed how even individual variation in average velocity alone is sufficient for a central-peripheral group structure to emerge, with faster moving individuals at the periphery of the group. In this model, individuals only differed in movement speed, not in fleeing frequency, and avoidance behavior was not employed. This suggests that a central-peripheral group structure can result from any behavioral mechanism that enhances differential average velocity in individuals.

A high fleeing frequency, frequent avoidance behavior and a high average velocity may be properties that are typical for subordinate individuals [37], [50], [101], [102]. By disentangling the contribution of each of these factors within a simulation model, we showed how each property independently results in peripheral spatial positions within a group, a venture that would be impossible in real groups of animals. Our results suggest a robust spatial group structure can be generated by several mechanisms simultaneously, which can be commonly found in primate groups.

### Avoidance behavior, aggressiveness and group spread

Two variables in the *avoidance model* directly influenced the degree of avoidance behavior: the minimum difference in dominance strength between two individuals that elicits avoidance behavior in the lower-ranking individual (*AV_DOM_DIFF*), and the spatial distance within which subordinates avoid potential aggressors (*AV_DIST*). In a group of primates there may be individual variation in the value of these parameters, depending on an individual's urge to avoid others. Different primate species may also differ in the degree of both overall and within-group variation of these variables, in relation to their degree of aggressiveness. Despotic species are characterized by a steeper dominance hierarchy and a higher variance of within-group aggressiveness. Within such a group, the urge to avoid dominants is higher and thus the dominance difference that elicits avoidance behavior (*AV_DOM_DIFF*) is expected to be lower within a group. Our results show that the lower this dominance difference (*AV_DOM_DIFF*), the more spatially spread out groups were. Similarly, subordinates in despotic species are expected to prefer to maintain a large distance to potential aggressors (*AV_DIST*). For avoidance at large distances our model also predicts a larger group spread. This suggests a possible mechanism for the larger group spread seen in groups of despotic animals compared to more egalitarian species, as has been shown in real primates [44], [103] and was suggested by other models [61], [104].

It has been suggested that avoidance behavior may be imperative in aggressive species that lack formal dominance signals [31], [36], [39], [42], [47] and our model predicts large group spread for such species. Researchers have found that groups of patas monkeys, a species lacking formal signals of submission, are much more spread out compared to species capable of formal submission [42], [47]. This may result from frequently employed avoidance behavior, as predicted by our model. In a species capable of formal submission, the urge to avoid is expected to be lower. Our model suggests such a group would be less spread out; however, the effects of dominance style and formal submission on spatial structure have yet to be explicitly formulated in a model.

### Avoidance behavior, aggressiveness and subgroup formation

Avoidance at a large distance (*AV_DIST* = 35 m) resulted in subgroups of avoiders and avoidees, which were spatially and socially distinct. In our model, the maximum group spread was restricted. Without this restriction, the group would have split into separate groups. Thus, in highly aggressive species, group split-up might occur as a result of frequent avoidance behavior among subgroups.

Modeling studies have shown how subgrouping patterns may emerge through foraging in a structured environment [105] or from affiliative bonds between the individuals [106]. Our model suggests another possible mechanism causing subgroup formation. High degrees of aggression and resulting avoidance behavior may be a major driving force behind subgroup formation and eventual group split-up, with subgroups of similar rank. Subgrouping patterns may thus simply be a consequence of aggression in the group. For example, Romero & Aureli [107] described two spatially distinct subgroups in a group of ring-tailed coatis, where aggression occurred more frequently between than within subgroups. Another study, in Barbary macaques [108], identified overt aggression between individuals of two subgroups as the main factor driving group split-up. Here, we present the first model that explicitly implemented and tested the effects of conflicts and spatial avoidance of aggressors on group structure, corroborating the organizing potential of social behavior.

Aggression resulting in spatial avoidance, however, may reduce group cohesion to suboptimal levels. Under such conditions, different behaviors that reduce the effect of aggression, but do not depend on an increase of distance, may evolve. Indeed many primates employ signals of submission, policing and post-conflict affiliation [24]–[31]. The effects of these alternative behaviors remain to be modeled.

In our model, we identified specific conditions leading to subgroup formation and eventual group split-up in our model system: 1) Individuals are socially attracted to group members. 2) There are subsets of individuals within a group, which do not avoid each other. Such a subset of individuals may form a subgroup. 3) When avoidance is employed at large distances between members of different subgroups, these subgroups may separate from each other spatially. The validity of these conditions needs to be tested in real primate groups.

We point out, that we did not aim to model all possible mechanisms of fission-fusion. Rather, our model shows how severe aggression and avoidance of potential aggressors may contribute to socio-spatial group patterns and subgroup formation. Moreover, the subgroups in our model assorted themselves according to dominance rank. Such a pattern has not been described for primate subgroups after group fission. This emphasizes the importance of kin and affiliative relationships in primate fission-fusion dynamics, especially with respect to subgroup composition. The composition of subgroups in our model seems to be similar to patterns observed in fish shoals. Many fish species assort themselves within the shoal according to size [109] and European minnows for example, are even able to recognize and avoid strong competitors within the shoal [110]. Although our model was inspired by primate behavior, it may be applicable more generally. Our model may explain how certain socio-spatial patterns may arise due to individual interactions in any species with a highly differentiated dominance hierarchy (or any other trait), given that the species is capable of individually recognizing this trait and responding with differentiated locomotion behavior.

### Encounter structure and spatial structure

Both the *fleeing model* and the *avoidance model* with avoidance at small distances (*AV_DIST* = 5 m) demonstrated (weak) centrality of dominant individuals. Moreover, in both of these models the dyadic distances between all group members were similar. Although the spatial patterning was similar, the models differed in the frequency and direction of encounters within the group. In the *fleeing model*, encounters were almost equally distributed among all possible dyads, whereas in the *avoidance model* more encounters took place among individuals of similar rank. This shows that social group properties, such as encounter structure, are not deducible from spatial relations alone.

### Conclusions

In this study we presented the group-level consequences of individual variation in movement properties. It has been shown that in some primate species group members vary in their degree of employing social vigilance: subordinates pay more attention to other group members than dominant individuals [48], [111], [112]. This variation in social information uptake may also give rise to group-level patterns, similar to the variation in movement properties presented in this paper. This will be further investigated in future models.

We could find no empirical studies in which avoidance behavior at a distance was observed. This is likely due to the difficulty of determining exactly which animal is avoided by another in a social group. Therefore, model studies can be of particular value, as they can serve as an informative tool to study the group-level consequences of such behavior, showing that difficult to observe behavior can have profound effects. This emphasizes the relevance to empirically study avoidance of aggressors in social groups.

We presented three models (*fleeing model*, *avoidance model* and *velocity model*), comparing different types of individual variation in movement characteristics within a group of model individuals. Using simulations, we assessed the effect of individual variation in fleeing tendency, in avoidance behavior and in velocity, to understand their effect on spatial and encounter structure. A central-peripheral group structure was found in all three investigated models, suggesting that any behavioral mechanism that selectively enhances movement differentiation in group members can be responsible for this specific spatial group structure, while the encounter structure is determined by the specific behavioral rules.

## Supporting Information

Figure S1 **Centrality of dominants in the** ***avoidance with fleeing-control model*** **.** This graph shows the relationship between an individual's dominance strength (myDOM) and its centrality (distance to the arithmetic center of the group in meters) for the *avoidance with fleeing-control model* (with different combinations of *AV_DOM_DIFF* (vertically, 0.2–0.4) and *AV_DIST* (horizontally, 5–35 m). Small distances to the arithmetic group center indicate more central positions. When the relation between dominance strength and centrality is steeper, centrality of dominants is more pronounced. For further implications of an individual's dominance strength depending on the model, see the Figure 4 legend. Boxplots show values of 50 simulation runs, averaged over time.
(TIF)

Figure S2 **Group spread in the** ***velocity model*** **.** This graph shows the group spread (in meters) in the *velocity model* for a range of values of *MAX_VELOCITY* (x-axis, 1–30 m/s). Boxplots show values of 50 simulation runs, averaged over time.
(TIF)

Figure S3 **Group spread in the** ***fleeing model*** **with different maximum turning angle.** This graph shows the group spread (in meters) in the *fleeing model* for a range of values for the maximum turning angle, used in the random walk procedure (x-axis, 180–90 degrees). Boxplots show values of 10 simulation runs, averaged over time.
(TIF)

Figure S4 **Centrality of dominants for maximum turning angle of 45 degrees.** This figure shows the relationship between an individual's dominance strength (myDOM) and its centrality (distance to the arithmetic center of the group in meters) for different models with a maximum turning angle of 45 degrees, as used in the random walk procedure. (A) *Fleeing model*. (B) *Avoidance model* (with different combinations of *AV_DOM_DIFF* (vertically, 0.2–0.4) and *AV_DIST* (horizontally, 5–35 m). Small distances to the arithmetic group center indicate more central positions. When the relation between dominance strength and centrality is steeper, centrality of dominants is more pronounced. For further implications of an individual's dominance strength depending on the model, see the Figure 4 legend. Boxplots show values of 10 simulation runs, averaged over time.
(TIF)

Figure S5 **Encounter structure in the probabilistic** ***avoidance model*** **with slope 30.** This figure shows the distribution and direction of encounters among the individuals of a group for the *avoidance model* with probabilistic avoidance with a slope of 30 for the avoidance chance function (with different combinations of *AV_DOM_DIFF* (vertically, 0.2–0.4) and *AV_DIST* (horizontally, 5–70 m). Encounters are directed from initiators (y-axis) to targets (x-axis), both are ordered by dominance strength (myDOM). For further implications of an individual's dominance strength depending on the model, see the Figure 4 legend. Plots show the mean values of 10 simulation runs. Dark shades represent frequent encounters. Values at the diagonal are by default not applicable.
(TIF)

Figure S6 **Centrality of dominants in the probabilistic** ***avoidance model*** **with slope 30.** This figure shows the relationship between an individual's dominance strength (myDOM) and its centrality (distance to the arithmetic center of the group in meters) for the *avoidance model* with probabilistic avoidance with a slope of 30 for the avoidance chance function (with different combinations of *AV_DOM_DIFF* (vertically, 0.2–0.4) and *AV_DIST* (horizontally, 5–70 m). Small distances to the arithmetic group center indicate more central positions. When the relation between dominance strength and centrality is steeper, centrality of dominants is more pronounced. For further implications of an individual's dominance strength depending on the model, see the Figure 4 legend. Boxplots show values of 10 simulation runs, averaged over time.
(TIF)

Figure S7 **Encounter structure in the probabilistic** ***avoidance model*** **with slope 5.** This figure shows the distribution and direction of encounters among the individuals of a group for the *avoidance model* with probabilistic avoidance with a slope of 30 for the avoidance chance function (with different combinations of *AV_DOM_DIFF* (vertically, 0.2–0.4) and *AV_DIST* (horizontally, 5–70 m). Encounters are directed from initiators (y-axis) to targets (x-axis), both are ordered by dominance strength (myDOM). For further implications of an individual's dominance strength depending on the model, see the Figure 4 legend. Plots show the mean values of 10 simulation runs. Dark shades represent frequent encounters. Values at the diagonal are by default not applicable.
(TIF)

Figure S8 **Snapshots of the socio-spatial group structure without restriction of fission.** This figure shows snapshots of the spatial composition of the group members for different models in which the restriction of the maximum group spread was switched off. (A) *Fleeing model*. (B) *Avoidance model* (with different combinations of *AV_DOM_DIFF* (vertically, 0.2–0.4) and *AV_DIST* (horizontally, 5–35 m). Shown is the total grid (300 by 300 meters) at one arbitrary point in time. Each arrowhead represents an individual. White shade represents a high dominance strength, dark shade represents a low dominance strength. The heading of an arrowhead represents the individual's visual orientation. For further implications of an individual's dominance strength depending on the model, see the Figure 4 legend.
(TIF)

Figure S9 **Group spread in the** ***fleeing model*** **with different values for fleeing distance.** This graph shows the group spread (in meters) in the *fleeing model* for a range of values of *FleeD* (x-axis, 1–20 m). Boxplots show values of 10 simulation runs, averaged over time.
(TIF)

Figure S10 **Centrality of dominants in the** ***fleeing model*** **with different values for fleeing distance.** This graph shows the relationship between an individual's dominance strength (myDOM) and its centrality (distance to the arithmetic center of the group in meters) for the *fleeing model* with different values of *FleeD* (horizontally, 1–20 m). Small distances to the arithmetic group center indicate more central positions. When the relation between dominance strength and centrality is steeper, centrality of dominants is more pronounced. For further implications of an individual's dominance strength depending on the model, see the Figure 4 legend. Boxplots show values of 10 simulation runs, averaged over time.
(TIF)

Figure S11 **Encounter structure in the** ***fleeing model*** **with different values for fleeing distance.** This figure shows the distribution and direction of encounters among the individuals of a group for the *fleeing model* with different values of *FleeD* (horizontally, 1–20 m). Encounters are directed from initiators (y-axis) to targets (x-axis), both are ordered by dominance strength (myDOM). For further implications of an individual's dominance strength depending on the model, see the Figure 4 legend. Plots show the mean values of 10 simulation runs. Dark shades represent frequent encounters. Values at the diagonal are by default not applicable.
(TIF)

Text S1 Win chance function and the distribution of fights among group members.
(PDF)

Text S2 Centrality of dominants as a model artifact.
(PDF)
