# Supplementary figures and images for: Better Safe than Sorry - Socio-Spatial Group Structure Emerges from Individual Variation in Fleeing, Avoidance or Velocity in an Agent-Based Model
Source: PLoS One. 2011 Nov 18;6(11):e26189. doi: 10.1371/journal.pone.0026189 (PMC3220670; doi:10.1371/journal.pone.0026189)

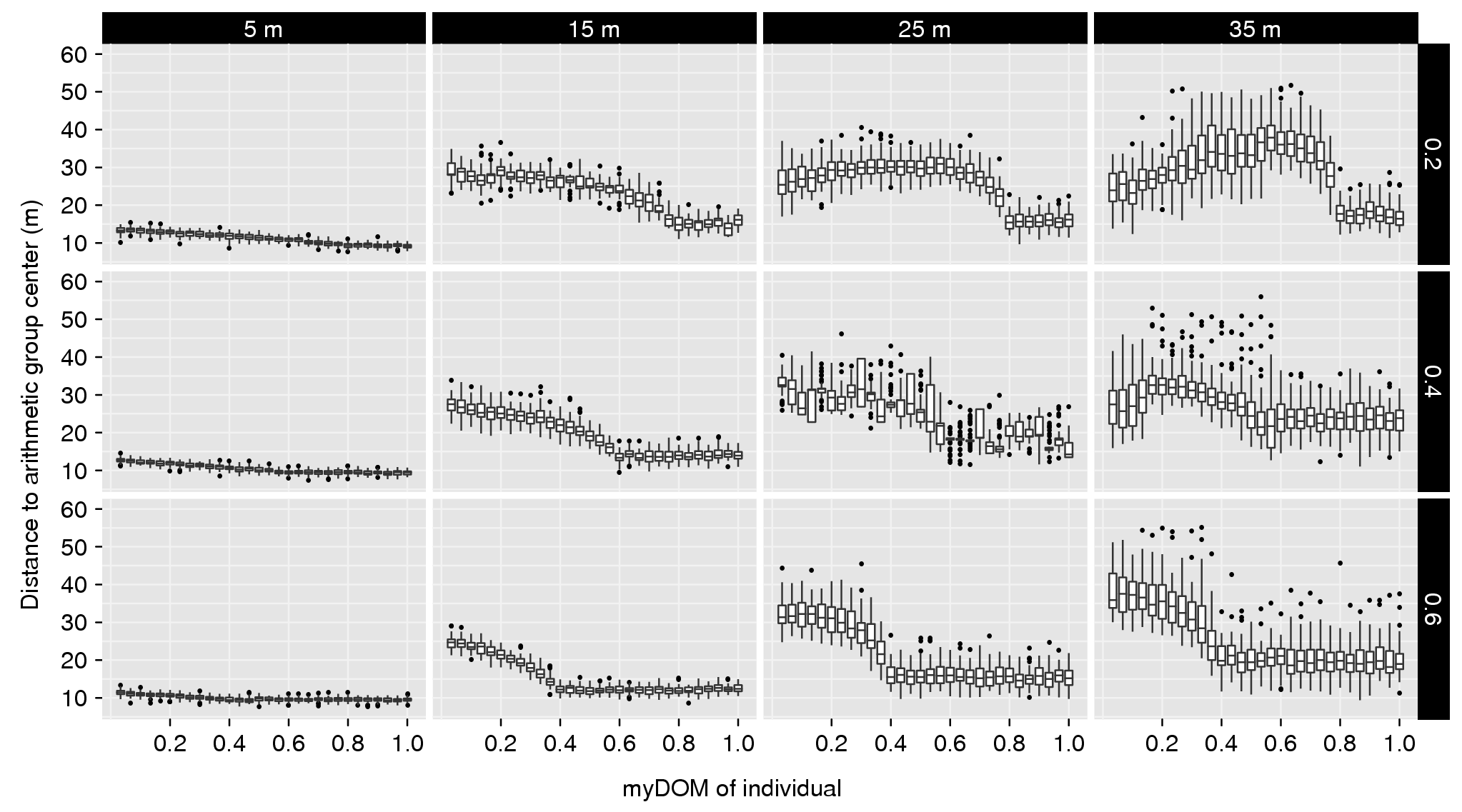

Supplement: Figure S1 — Centrality of dominants in the avoidance with fleeing-control model . This graph shows the relationship between an individual's dominance strength (myDOM) and its centrality (distance to the arithmetic center of the group in meters) for the avoidance with fleeing-control model (with different combinations of AV_DOM_DIFF (vertically, 0.2–0.4) and AV_DIST (horizontally, 5–35 m). Small distances to the arithmetic group center indicate more central positions. When the relation between dominance strength and centrality is steeper, centrality of dominants is more pronounced. For further implications of an individual's dominance strength depending on the model, see the Figure 4 legend. Boxplots show values of 50 simulation runs, averaged over time. (TIF) [file pone.0026189.s001.tif]

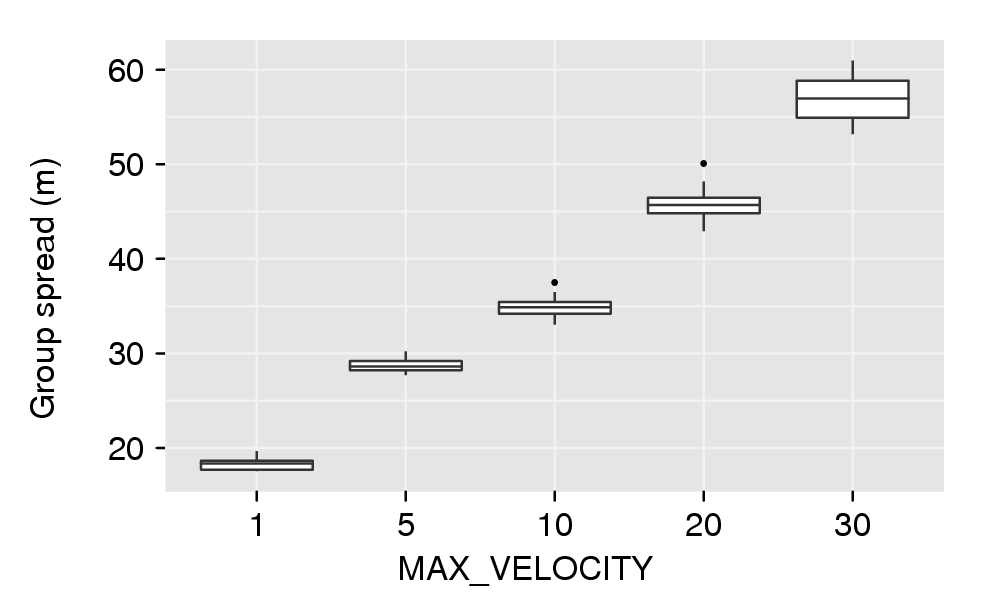

Supplement: Figure S2 — Group spread in the velocity model . This graph shows the group spread (in meters) in the velocity model for a range of values of MAX_VELOCITY (x-axis, 1–30 m/s). Boxplots show values of 50 simulation runs, averaged over time. (TIF) [file pone.0026189.s002.tif]

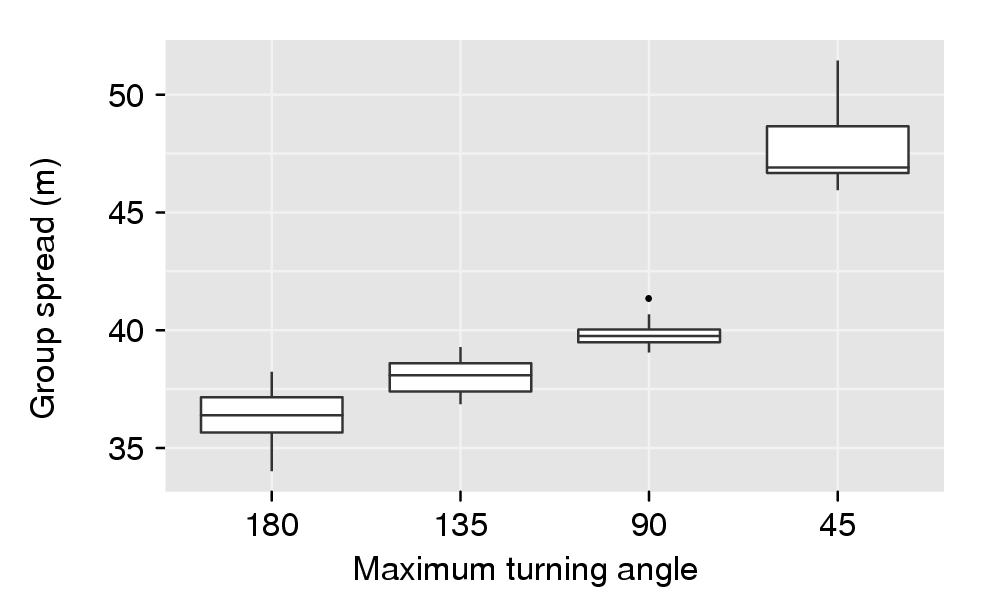

Supplement: Figure S3 — Group spread in the fleeing model with different maximum turning angle. This graph shows the group spread (in meters) in the fleeing model for a range of values for the maximum turning angle, used in the random walk procedure (x-axis, 180–90 degrees). Boxplots show values of 10 simulation runs, averaged over time. (TIF) [file pone.0026189.s003.tif]

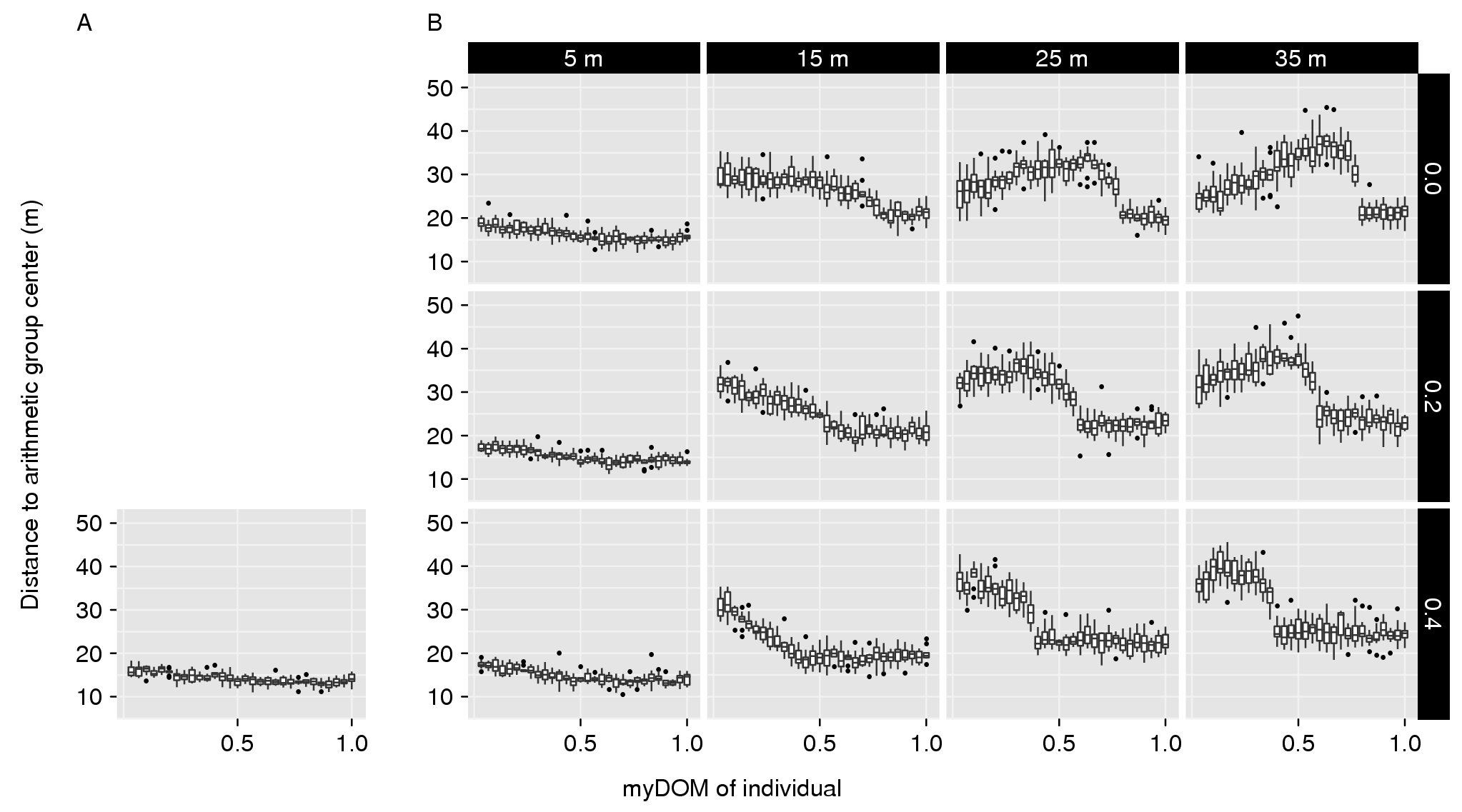

Supplement: Figure S4 — Centrality of dominants for maximum turning angle of 45 degrees. This figure shows the relationship between an individual's dominance strength (myDOM) and its centrality (distance to the arithmetic center of the group in meters) for different models with a maximum turning angle of 45 degrees, as used in the random walk procedure. (A) Fleeing model. (B) Avoidance model (with different combinations of AV_DOM_DIFF (vertically, 0.2–0.4) and AV_DIST (horizontally, 5–35 m). Small distances to the arithmetic group center indicate more central positions. When the relation between dominance strength and centrality is steeper, centrality of dominants is more pronounced. For further implications of an individual's dominance strength depending on the model, see the Figure 4 legend. Boxplots show values of 10 simulation runs, averaged over time. (TIF) [file pone.0026189.s004.tif]

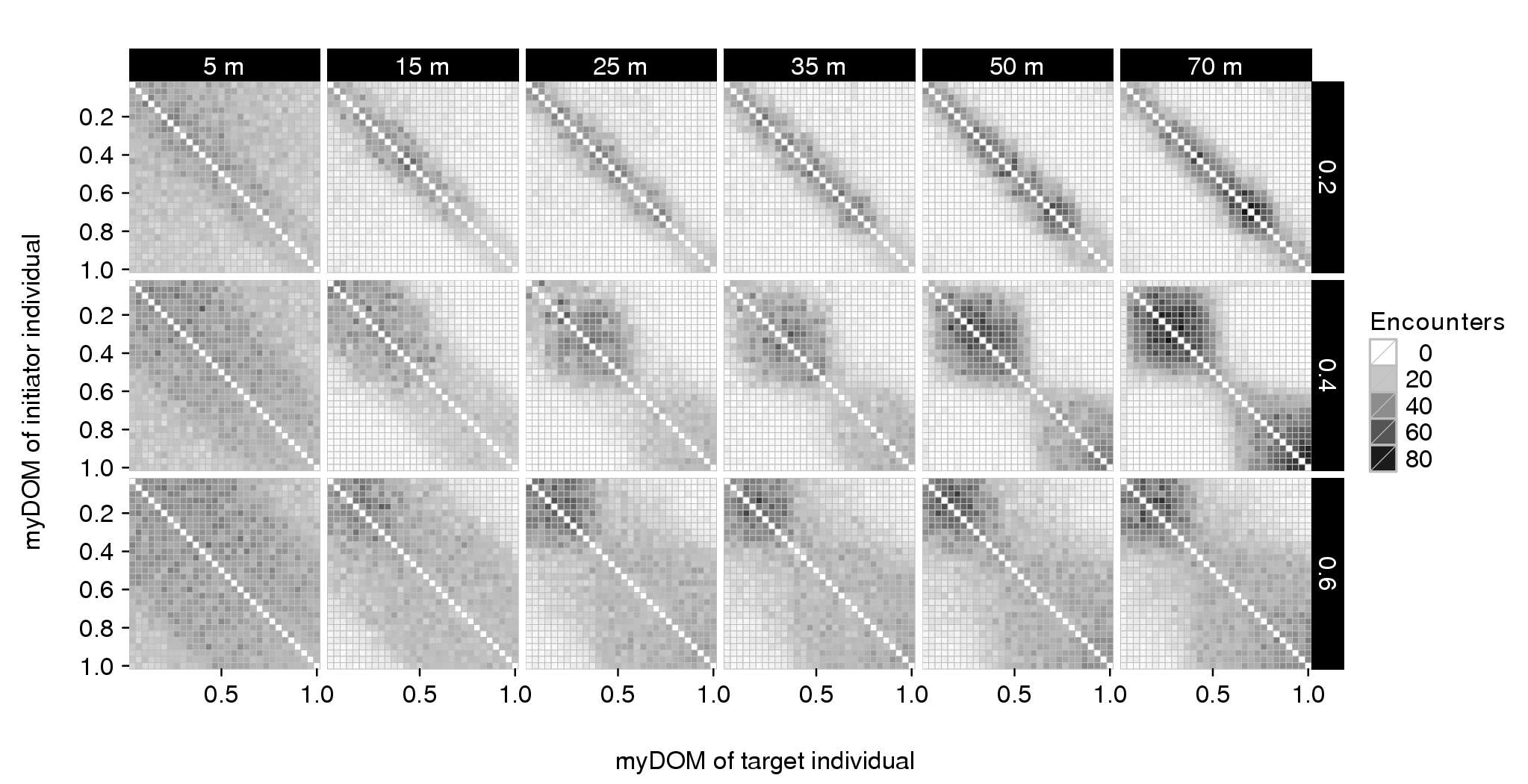

Supplement: Figure S5 — Encounter structure in the probabilistic avoidance model with slope 30. This figure shows the distribution and direction of encounters among the individuals of a group for the avoidance model with probabilistic avoidance with a slope of 30 for the avoidance chance function (with different combinations of AV_DOM_DIFF (vertically, 0.2–0.4) and AV_DIST (horizontally, 5–70 m). Encounters are directed from initiators (y-axis) to targets (x-axis), both are ordered by dominance strength (myDOM). For further implications of an individual's dominance strength depending on the model, see the Figure 4 legend. Plots show the mean values of 10 simulation runs. Dark shades represent frequent encounters. Values at the diagonal are by default not applicable. (TIF) [file pone.0026189.s005.tif]

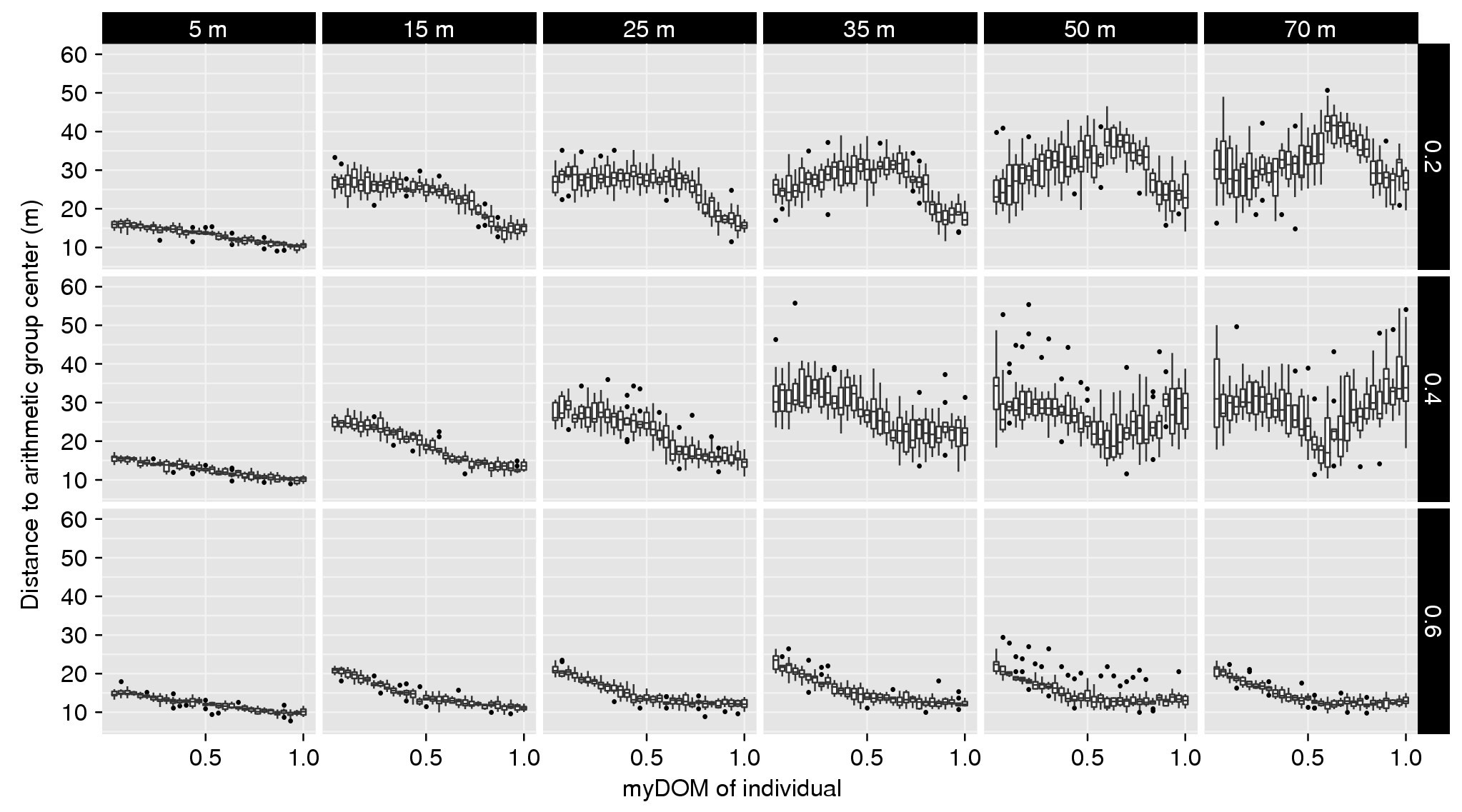

Supplement: Figure S6 — Centrality of dominants in the probabilistic avoidance model with slope 30. This figure shows the relationship between an individual's dominance strength (myDOM) and its centrality (distance to the arithmetic center of the group in meters) for the avoidance model with probabilistic avoidance with a slope of 30 for the avoidance chance function (with different combinations of AV_DOM_DIFF (vertically, 0.2–0.4) and AV_DIST (horizontally, 5–70 m). Small distances to the arithmetic group center indicate more central positions. When the relation between dominance strength and centrality is steeper, centrality of dominants is more pronounced. For further implications of an individual's dominance strength depending on the model, see the Figure 4 legend. Boxplots show values of 10 simulation runs, averaged over time. (TIF) [file pone.0026189.s006.tif]

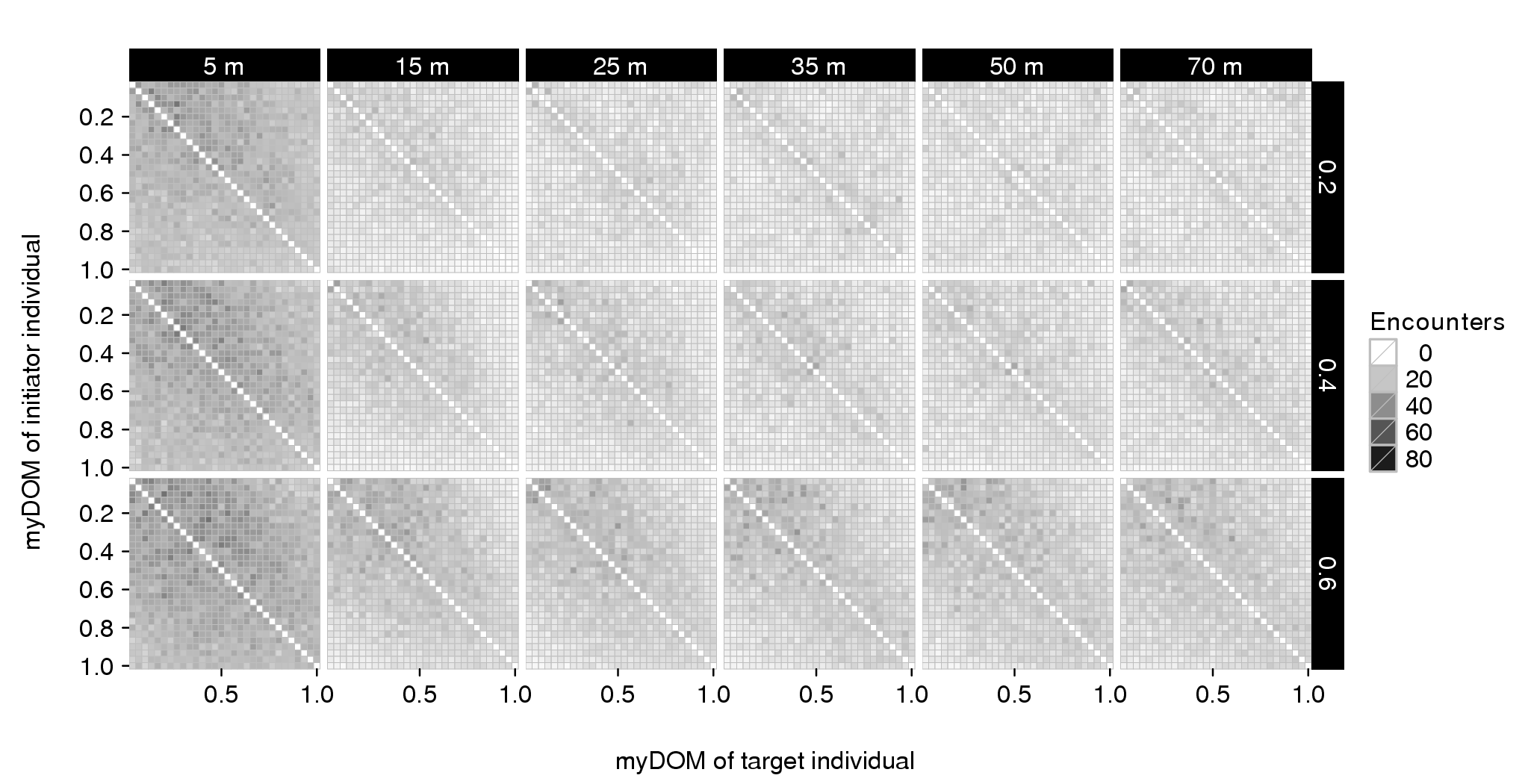

Supplement: Figure S7 — Encounter structure in the probabilistic avoidance model with slope 5. This figure shows the distribution and direction of encounters among the individuals of a group for the avoidance model with probabilistic avoidance with a slope of 30 for the avoidance chance function (with different combinations of AV_DOM_DIFF (vertically, 0.2–0.4) and AV_DIST (horizontally, 5–70 m). Encounters are directed from initiators (y-axis) to targets (x-axis), both are ordered by dominance strength (myDOM). For further implications of an individual's dominance strength depending on the model, see the Figure 4 legend. Plots show the mean values of 10 simulation runs. Dark shades represent frequent encounters. Values at the diagonal are by default not applicable. (TIF) [file pone.0026189.s007.tif]

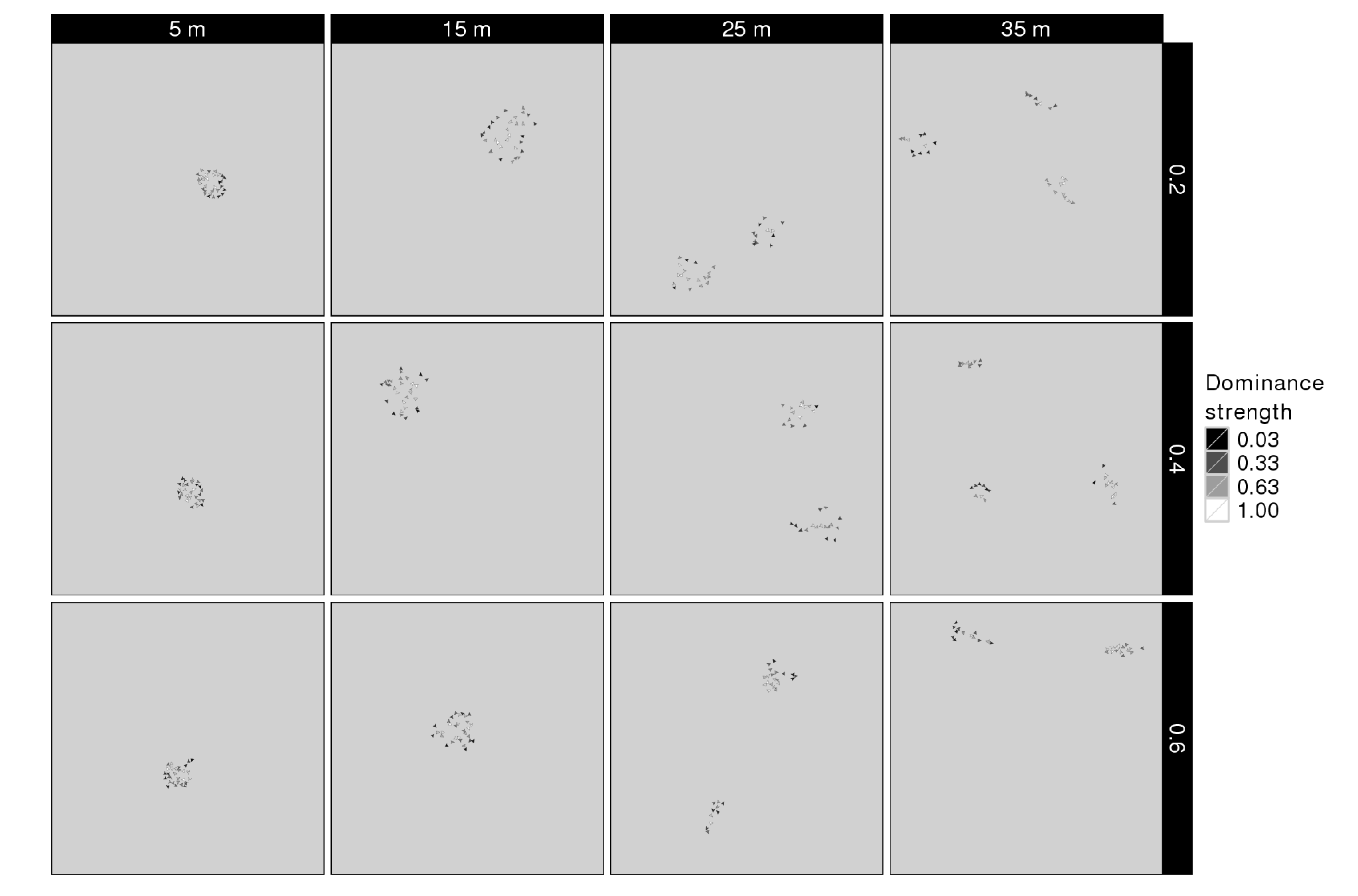

Supplement: Figure S8 — Snapshots of the socio-spatial group structure without restriction of fission. This figure shows snapshots of the spatial composition of the group members for different models in which the restriction of the maximum group spread was switched off. (A) Fleeing model. (B) Avoidance model (with different combinations of AV_DOM_DIFF (vertically, 0.2–0.4) and AV_DIST (horizontally, 5–35 m). Shown is the total grid (300 by 300 meters) at one arbitrary point in time. Each arrowhead represents an individual. White shade represents a high dominance strength, dark shade represents a low dominance strength. The heading of an arrowhead represents the individual's visual orientation. For further implications of an individual's dominance strength depending on the model, see the Figure 4 legend. (TIF) [file pone.0026189.s008.tif]

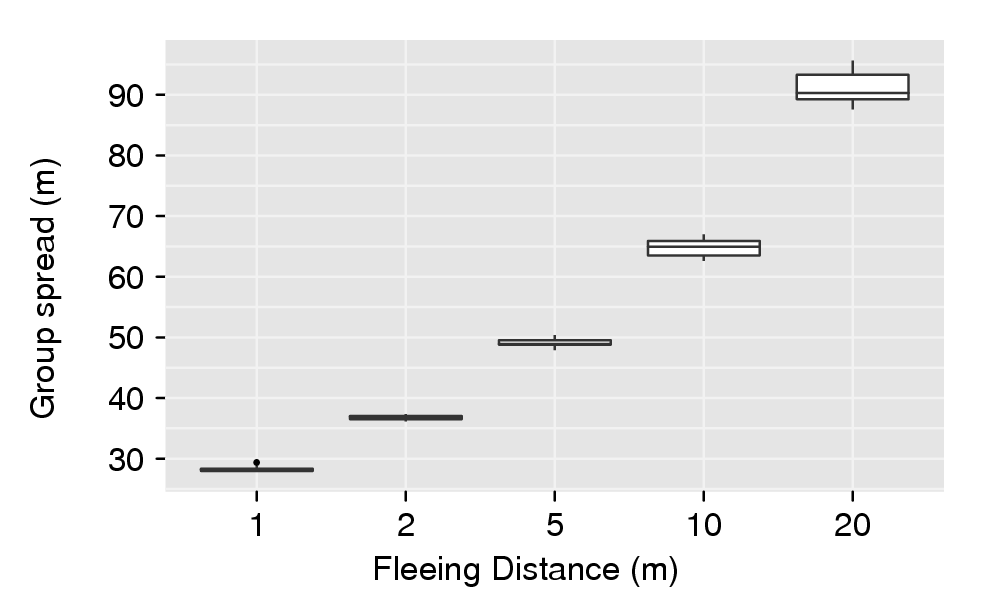

Supplement: Figure S9 — Group spread in the fleeing model with different values for fleeing distance. This graph shows the group spread (in meters) in the fleeing model for a range of values of FleeD (x-axis, 1–20 m). Boxplots show values of 10 simulation runs, averaged over time. (TIF) [file pone.0026189.s009.tif]

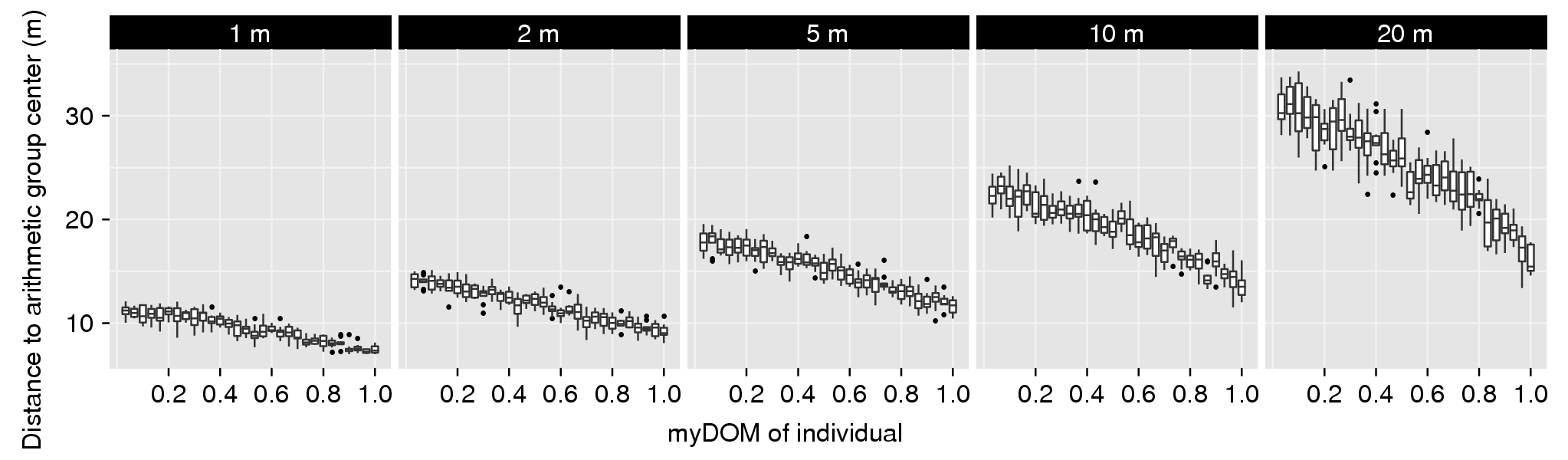

Supplement: Figure S10 — Centrality of dominants in the fleeing model with different values for fleeing distance. This graph shows the relationship between an individual's dominance strength (myDOM) and its centrality (distance to the arithmetic center of the group in meters) for the fleeing model with different values of FleeD (horizontally, 1–20 m). Small distances to the arithmetic group center indicate more central positions. When the relation between dominance strength and centrality is steeper, centrality of dominants is more pronounced. For further implications of an individual's dominance strength depending on the model, see the Figure 4 legend. Boxplots show values of 10 simulation runs, averaged over time. (TIF) [file pone.0026189.s010.tif]

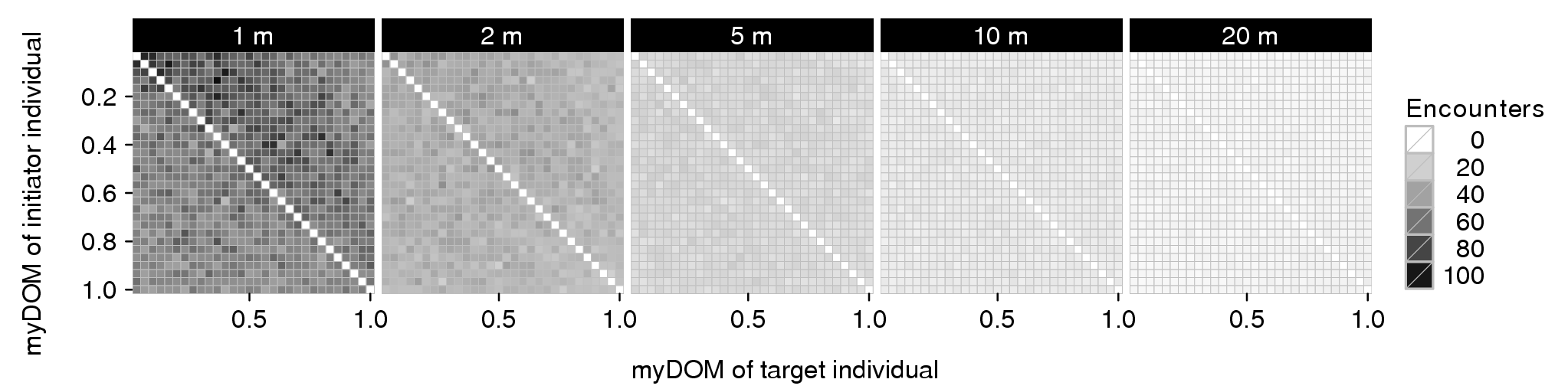

Supplement: Figure S11 — Encounter structure in the fleeing model with different values for fleeing distance. This figure shows the distribution and direction of encounters among the individuals of a group for the fleeing model with different values of FleeD (horizontally, 1–20 m). Encounters are directed from initiators (y-axis) to targets (x-axis), both are ordered by dominance strength (myDOM). For further implications of an individual's dominance strength depending on the model, see the Figure 4 legend. Plots show the mean values of 10 simulation runs. Dark shades represent frequent encounters. Values at the diagonal are by default not applicable. (TIF) [file pone.0026189.s011.tif]
