# Supplementary material for: Better Safe than Sorry - Socio-Spatial Group Structure Emerges from Individual Variation in Fleeing, Avoidance or Velocity in an Agent-Based Model
Source: PLoS One. 2011 Nov 18;6(11):e26189. doi: 10.1371/journal.pone.0026189 (PMC3220670; doi:10.1371/journal.pone.0026189)
Supplement: Text S1 — Win chance function and the distribution of fights among group members. (PDF) [file pone.0026189.s012.pdf]

Win chance function and the distribution of fights among group members

In our model, an individual's chance of starting a fight depends on its win chance against its opponent. In contrast to the sigmoidal win chance we used here, Hemelrijk implemented a relative win chance in her DomWorld model [1]. With this relative win chance, individual A's chance of winning from individual B gets calculated as follows:

$$w_{AB} = \frac{myDOM_A}{myDOM_A + myDOM_B}, \tag{1}$$

i.e. the win chance of an individual depends on the relative difference in dominance compared to the opponent. This has been criticized elsewhere [2], because of resulting unrealistically high dominance updates in low-ranking individuals after a fight. Furthermore, with a relative win chance low-ranking individuals with a certain dominance difference differ much more in their win chances than two higher-ranking opponents with the same dominance difference (Table S1). On the other hand, when applying a sigmoidal win chance (as suggested by de Vries [2]), win chance is the same whenever dominance difference is the same.

We found that the function of the win chance function may crucially affect the number of fights within a group and especially how fights are distributed among members of the group. We compared three models with different functions for the win chance: relative (as in Hemelrijk [1]), sigmoidal (as was used in the current paper, Equation 3) and absolute win chance. The latter is similar to the sigmoidal win chance, except that the curve is much steeper and thus approximating a step function. To get an absolute win chance we simply set the parameter η (Equation 3) to $(60/MAX\_DOM)$, instead of $(6/MAX\_DOM)$ for the sigmoidal win chance.

With a relative win chance function, almost all dyads engage in fights regularly (Figure 1A). Only the lowest-ranking individuals restrict fights to opponents of similar rank. On the other hand, with a sigmoidal win chance, escalated fights are restricted to opponents of similar rank (Figure 1B). With an absolute win chance, fights are even more restricted to the diagonal (Figure 1C).

Note that these effects are not due to differences in group spread or encounter rates (see Table 2). The encounter rates in Figure S1 comply with the chance of starting a fight after encounter for each

possible dyad. The chance that a certain dyad engages in a fight after encounter can simply be calculated as the product of both individuals' win chances, as in our model an actual fight takes place only if both opponents agree to a fight (see Figure 3). Therefore the calculated fight chances are also symmetrical for each dyad.

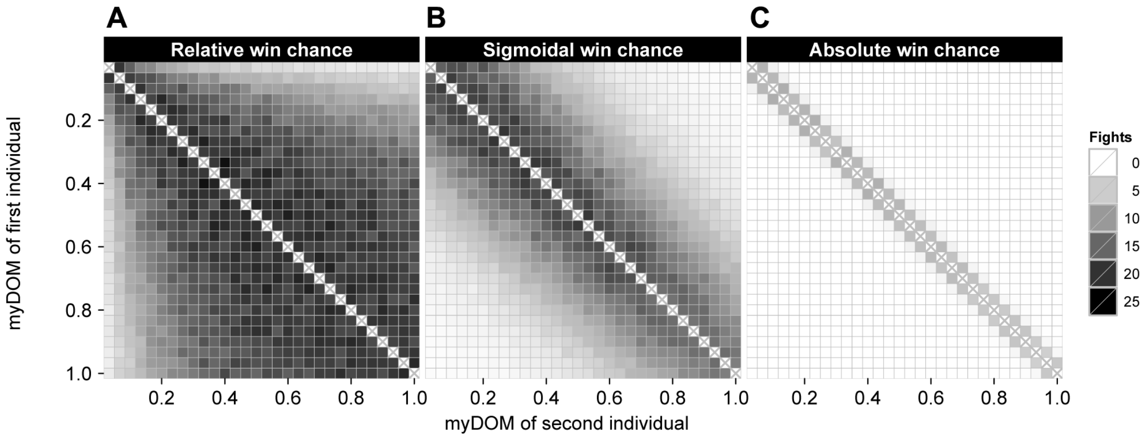

Figure 1. Fight structure for different win chance functions. This figure shows the distribution of fights among members of a group of individuals for the fleeing model with three different win chance functions: (A) Relative win chance. (B) Sigmoidal win chance. (C) Absolute win chance. Numbers of fights are symmetrical for dyads, as both opponents have to agree to engage in a fight. Plots show the mean values of 50 simulation runs. Dark shades represent frequent fights. Values at the diagonal (x) are by default not applicable.

Table 1. Differences of the relative and sigmoidal win chance functions for pairs with same dominance distance.

$myDOM_A$	$myDOM_B$	Dominance difference	Relative w_{AB}	Relative w_{BA}	Sigmoidal w_{AB}	Sigmoidal w_{BA}
0.1	0.2	0.1	0.33	0.66	0.35	0.65
0.9	1.0	0.1	0.47	0.53	0.35	0.65

Table 2. Group spread and average number of encounters per dyad, for different win chance functions.

**Win chance:**	**Relative**	**Sigmoidal**	**Absolute**
Group spread	34.9 ± 0.6 m	36.5 ± 0.7 m	38.7 ± 0.7 m
Average number of encounters per possible dyad	37.9 ± 0.6	37.8 ± 0.9	37.7 ± 1.2

Values are means ± standard deviation of the group average (N = 50 simulation runs).

References

1. Hemelrijk CK (1998) Spatial centrality of dominants without positional preference. In: Artificial Life VI: Proceedings of the Sixth International Conference on Artificial Life. Cambridge, Mass: MIT Press, pp. 307-315.

2. de Vries H (2009) On using the DomWorld model to evaluate dominance ranking methods. Behaviour 146: 843–869.
