# Supplementary material for: Better Safe than Sorry - Socio-Spatial Group Structure Emerges from Individual Variation in Fleeing, Avoidance or Velocity in an Agent-Based Model
Source: PLoS One. 2011 Nov 18;6(11):e26189. doi: 10.1371/journal.pone.0026189 (PMC3220670; doi:10.1371/journal.pone.0026189)
Supplement: Text S2 — Centrality of dominants as a model artifact. (PDF) [file pone.0026189.s013.pdf]

Centrality of dominants as a model artifact

In the original DomWorld model [1] as well as in our model, individuals engage in dominance interactions. After a fight, the loser flees from the winner and the winner chases the loser. Furthermore, in the DomWorld model (but not in our model here) the winner usually turns about a certain angle after chasing. This turning, or "wiggling", was implemented by Hemelrijk to (artificially) prevent too many repeated interactions between the same two opponents [1].

Our model and the DomWorld model show that the fleeing behavior of subordinates after a fight shapes a central-peripheral spatial group structure. However, an earlier replication of the DomWorld model did not find this spatial pattern after excluding the chasing behavior of the winner after a fight (see Appendix of Bryson *et al.* [2]). This was quite unexpected as distances between loser and winner should increase even more than when chasing behavior is excluded from the model, this in turn should result in a more pronounced spatial structure.

After extensive examination of this case, we found that this change in spatial pattern was due to two main factors: first, individuals always engaged in an interaction when they encountered each other and second when chasing was excluded, this automatically excluded subsequent wiggling of the winner.

When individuals interact in Bryson *et al.*'s model, both opponents orient towards each other. After the loser flees, the winner is still oriented towards the loser. When chasing is included in the model, central dominants usually win and chase losers towards the periphery. However if the winner wiggles about a considerable angle, its visual orientation is directed away from its former opponent. This not only decreases the chance of renewed encounters with this same individual, it also increases the chance of perceiving others near the group center, thereby ensuring movement back towards the group center. Through this, the winner returns to the group center earlier than the loser, who cannot turn around towards the group until its next activation. In other words, wiggling gives the winner a head start in returning to the group center, while the loser has to "skip a turn". This effect is even more exaggerated, when the individual's movement is straight instead of a random walk. The loser can now only turn back towards the group after it is perceiving too few others in $NEAR\_DIST$ anymore, i.e. after it reached the group periphery.

We tested a range of values for the wiggle-angle in a simplified version of our fleeing model, where individuals always fight on encounter (as in the model in the Appendix of Bryson *et al.*). A large wiggle-

angle resulted in a more pronounced central-peripheral group structure (Figure 1). In contrast, when the wiggle-angle was small or zero, the winner of a fight was still oriented towards the loser and presumably engaged in further interactions with the loser. This results in less central winners (dominants) compared to a model with large wiggling.

Interestingly, the width of the wiggle-angle does not have this effect when encountering individuals do not instantaneously engage in a fight. In the DomWorld model and in our model, individuals can decide whether to fight or to flee. As a consequence, a large number of encounters resulted in fleeing behavior without an interaction taking place and before both opponents could orient towards each other. Dominants are left at the group center and did not always chase or follow the subordinates. Thus, whether winners wiggle after chasing does not have such an effect on the group structure in our model.

This elaboration of the wiggle-angle illustrates how each rule that is put into a behavioral model should preferably be motivated by the actual behavior observed in real animals. Furthermore, as far as it is feasible, the effect of each rule should be analyzed thoroughly to prevent model artifacts distorting the explanatory potential of a model.

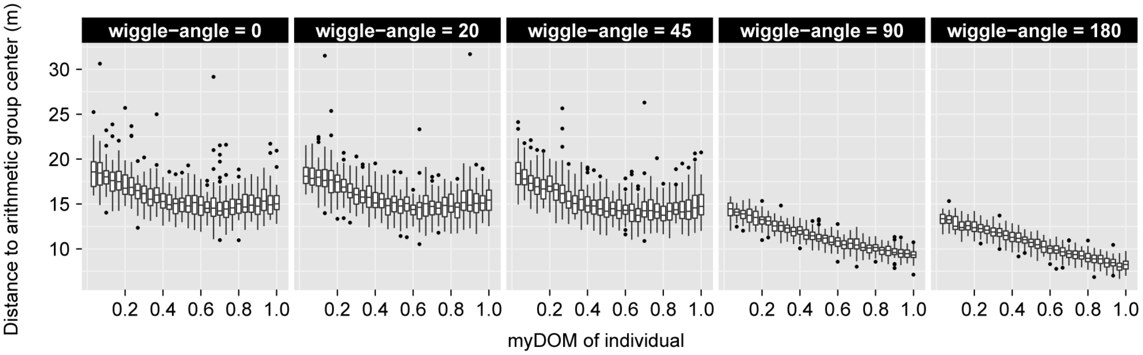

Figure 1. Centrality of dominants for different wiggle-angles. This graph shows the relationship between an individual's distance to the arithmetic center of the group (in meters) and their dominance strength in the fleeing model for a range of values of wiggle-angle: 0, 20, 45, 90 and 180 degrees. Wiggle-angle is the angle (in degrees) a winner turns away from its opponent after chasing it. Boxplots show values of 10 simulation runs, averaged over time.

References

1. Hemelrijk CK (1998) Spatial centrality of dominants without positional preference. In: Artificial Life VI: Proceedings of the Sixth International Conference on Artificial Life. Cambridge, Mass: MIT Press, pp. 307-315.

2. Bryson JJ, Ando Y, Lehmann H (2007) Agent-based modelling as scientific method: a case study analysing primate social behaviour. Philosophical Transactions of the Royal Society B: Biological Sciences 362: 1685-1699.
